# Multimetal Doping and Heterostructure Engineering of RuO_2_ for Durable and Efficient Oxygen Evolution

**DOI:** 10.1002/smsc.202500546

**Published:** 2025-12-24

**Authors:** Md. Mofakkharulhashan, Shiqi Wang, Hugo L. S. Santos, Mykhailo Chundak, Mikko Ritala, Pedro H. C. Camargo

**Affiliations:** ^1^ Department of Chemistry University of Helsinki A.I. Virtasen aukio 1 FIN‐0014 Helsinki Finland

**Keywords:** electrocatalysis, multimetal doping, oxygen evolution reaction, RuO_2_ heterostructures, water electrolysis

## Abstract

The oxygen evolution reaction (OER) is the primary kinetic bottleneck in water electrolysis, requiring catalysts that are both efficient and durable. Here, a MnCoNi–RuO_2_ (MCN–RuO_2_) heterostructured catalyst synthesized via a controlled impregnation–annealing–etching process that integrates multimetal doping with mixed‐phase oxide formation is reported. Structural analyses reveal a RuO_2_ host lattice interfaced with MnO and spinel‐type CoNiO_*x*_ domains, generating lattice distortion, oxygen vacancies, and defect‐rich interfaces that tune the electronic structure and enrich active sites. Electrochemical tests demonstrate overpotentials as low as 200 mV at 10 mA cm^−2^, a low Tafel slope, and markedly improved stability relative to commercial RuO_2_ and IrO_2_. The catalyst also retains high activity under acidic conditions and, when implemented in an anion exchange membrane water electrolyzer, sustains industrially relevant operation for 100 h with minimal degradation. Density functional theory calculations reveal that multimetal incorporation drives charge redistribution, lowers the work function, and shifts Ru‐4d states, reducing the barrier for the rate‐determining *O → *OOH step while enhancing stability against Ru dissolution. These findings establish MCN–RuO_2_ as a versatile, Ir‐free platform and demonstrate multimetal doping with heterointerface engineering as a powerful strategy for designing next‐generation OER catalysts.

## Graphical abstract

A MnCoNi–RuO_2_ heterostructure engineered through multimetal doping and mixed‐oxide interface formation generates lattice distortion, oxygen vacancies, and electronically optimized active sites for the oxygen evolution reaction. MnCoNi–RuO_2_ achieves high activity and durability across alkaline and acidic media, sustaining 100 h (AEMWE) anion exchange membrane alkaline electrolysis operation. Density functional theory reveals charge redistribution and lowered *O → *OOH barriers driving electrocatalytic performance.

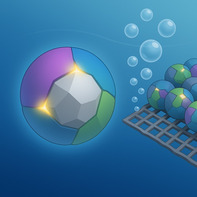

## 1 Introduction

The accelerating demand for renewable energy storage and conversion technologies has placed water electrolysis at the forefront of sustainable hydrogen production.^[^ ^1^ ^]^ Among the two half‐reactions, the oxygen evolution reaction (OER) is the primary kinetic bottleneck, requiring highly efficient and durable electrocatalysts to sustain operation in both acidic and alkaline environments.^[^ ^2^ ^]^ Ruthenium dioxide (RuO_2_) and iridium dioxide (IrO_2_) are widely regarded as benchmark catalysts due to their favorable intrinsic activity.^[^ ^3^, ^4^, ^5^, ^6^ ^]^ However, the scarcity and cost of iridium, together with the limited long‐term stability of RuO_2_ under harsh electrochemical conditions, have spurred intense efforts to design iridium‐free alternatives.^[^ ^7^ ^]^ Recent advances highlight the potential of defect engineering, multimetal doping, and heterostructure formation to tailor electronic states, stabilize active sites, and optimize adsorption energetics for OER catalysis.^[^ ^8^, ^9^, ^10^, ^11^, ^12^, ^13^ ^]^

Despite these developments, achieving catalysts that simultaneously combine high intrinsic activity, structural robustness, and applicability across different pH conditions remains challenging.^[^ ^14^ ^]^ While single‐element doping strategies can modulate electronic states or introduce beneficial defects, their impact is often modest and strongly dependent on dopant–host compatibility, underscoring the need for integrated multimetal approaches that enable cooperative electronic and structural effects.^[^ ^8^, ^15^ ^]^ Likewise, heterostructured oxides offer opportunities for interfacial synergy, but controlling phase interactions at the atomic scale is difficult.^[^ ^16^ ^]^ Overcoming these barriers requires synthetic strategies that deliberately integrate multimetal incorporation with controlled structural modulation to unlock cooperative effects, balancing conductivity, stability, and catalytic activity.

Here, we report a MCN–RuO_2_ catalyst synthesized via a controlled impregnation–annealing–etching strategy that combines multimetal doping with phase‐segregated oxide formation. This design produces a RuO_2_ matrix with lattice distortion, secondary oxide domains, and defect‐rich interfaces that enhance conductivity and enrich active sites. The formation of phase‐separated oxide domains and heterointerfaces in the resulting multimetal system was verified experimentally using selected‐area electron diffraction (SAED), high‐resolution transmission electron microscopy (HRTEM), and X‐ray diffraction (XRD). Electrochemical measurements reveal high OER activity and durability, with overpotentials as low as 200 mV at 10 mA cm^−2^ and superior long‐term stability compared to commercial RuO_2_ and IrO_2_. Importantly, the catalyst demonstrates robust performance in both alkaline and acidic electrolytes and sustains industrially relevant operation in anion exchange membrane electrolyzers. Complementary density functional theory (DFT) calculations attribute these enhancements to dopant‐induced charge redistribution, d‐band modulation, and interfacial stabilization of key intermediates. Together, these results establish a generalizable design pathway for iridium‐free, multimetal‐doped heterostructures, advancing the development of scalable OER catalysts for sustainable hydrogen production.

## 2 Results and Discussion

### 2.1 Synthesis Rationale and Material Characterization

The design of advanced OER catalysts requires a balance between activity, stability, and cost, and this balance can only be achieved through deliberate selection of elements and architectures rather than random compositional mixing. In this work, Mn, Co, and Ni were chosen as first‐row transition‐metal dopants to strategically modulate the RuO_2_ host. Each of these elements has been individually reported to promote lattice distortion, create oxygen vacancies, or tune the electronic structure in ways that are beneficial for OER.^[^ ^17^, ^18^, ^19^ ^]^ Their simultaneous incorporation, however, offers the opportunity to integrate multiple effects into a single catalyst: Mn contributes redox flexibility and vacancy formation,^[^ ^18^ ^]^ Co enhances conductivity and stabilizes intermediate binding,^[^ ^20^ ^]^ and Ni fine‐tunes the d‐band center and supports long‐term durability.^[^ ^7^, ^21^ ^]^ Embedding these dopants within a RuO_2_ matrix is expected not only to mitigate the intrinsic stability challenges of Ru‐based catalysts but also to leverage heterointerfaces formed with segregated oxide phases, thereby enriching the number and nature of active sites.^[^ ^22^, ^23^ ^]^

Guided by this rationale, we developed a controlled impregnation–annealing–etching approach^[^ ^24^ ^]^ to prepare a MCN–RuO_2_ heterostructure to combine electronic modulation, structural stability, and interfacial synergy for enhanced OER performance. The catalyst was synthesized via a three‐step process. In the first step, a wet impregnation method was used to disperse the metal precursors onto a carbon vulcan support, with ultrasonication ensuring uniform distribution of the metal species. The carbon matrix served to prevent premature aggregation, enabling the formation of a homogeneous precursor distribution. In the second step, the metal precursor mixture in carbon was annealed in air, converting the precursors into their corresponding oxides while simultaneously removing the carbon support. This thermal treatment was carefully tuned to promote crystallization and structural integrity of the resulting oxides. Finally, an acid leaching step selectively removed any unstable species, leading to the formation of the MCN–RuO_2_ nanoparticles. The importance of this etching treatment was confirmed electrochemically: as shown in Figure S1, Supporting Information, the etched catalyst delivered substantially higher current density and lower onset potential compared to the nonetched sample. This enhancement can be attributed to the removal of inactive or unstable surface oxides, which exposes more catalytically accessible sites and improves charge transfer across the electrode–electrolyte interface. Thus, the etching process is essential for activating the catalyst for optimal OER performance.^[^ ^25^ ^]^

After the impregnation steps, the samples were calcined to generate the final MCN–RuO_2_ nanoparticles. To optimize calcination conditions, the MCN–RuO_2_ samples were annealed at temperatures ranging from 350 to 650 °C in air. Electrochemical screening revealed that the catalyst treated at 450 °C exhibited the best OER activity (Figure S2, Supporting Information). It is plausible that moderate calcination promotes the formation of highly dispersed oxide phases while suppressing excessive particle size growth or phase segregation. Based on these findings, the 450 °C sample was selected for detailed structural and morphological analysis.

**Figure 1:**
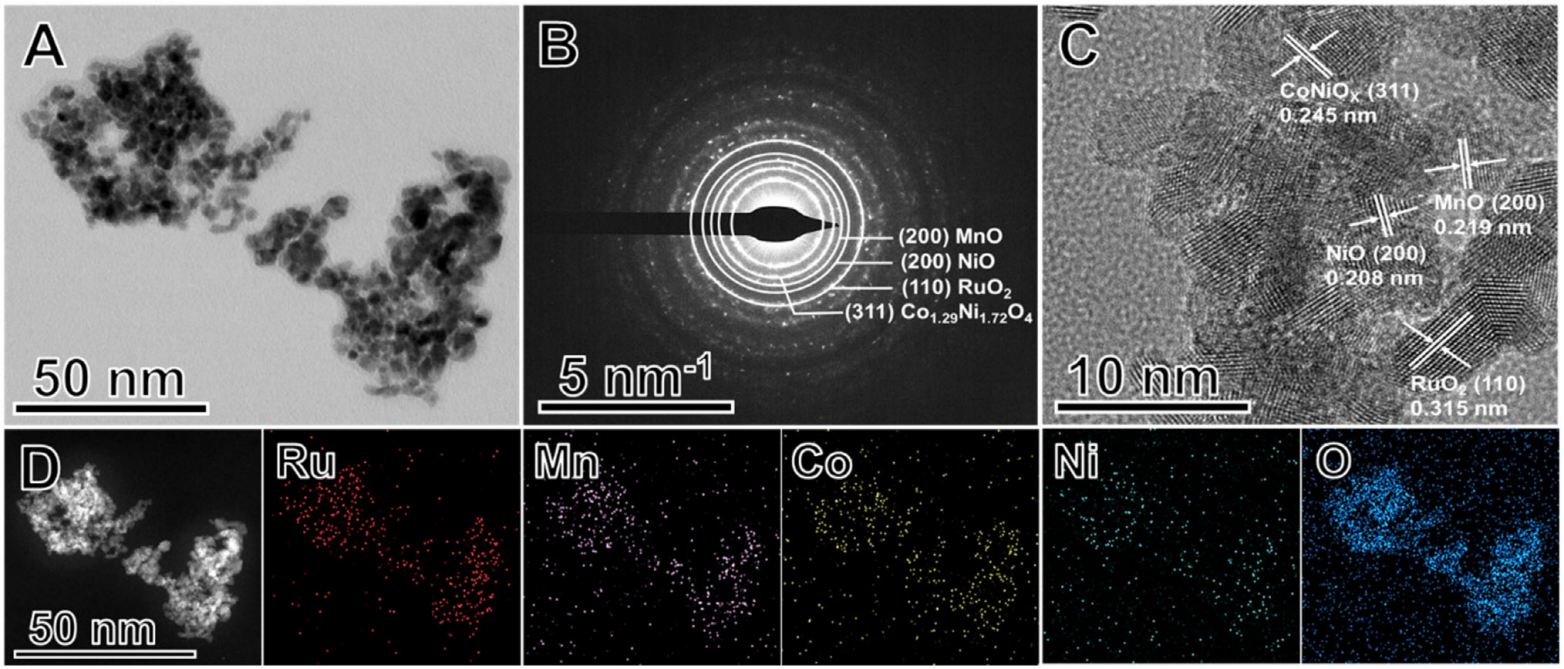
Morphological and structural characterization of MCN–RuO_2_. A) TEM image showing relatively uniform with sizes <5 nm. B) SAED pattern displaying diffraction rings indexed to multiple crystalline phases, including RuO_2_ (110), Co_1.29_Ni_1.72_O_4_ (311), MnO (200), and NiO (200). C) HRTEM image highlighting lattice fringes with interplanar spacings of 0.315 and 0.308 nm corresponding to RuO_2_ (110), 0.219 nm corresponding to MnO (200), 0.208 nm corresponding to NiO (200), and 0.245 nm corresponding to Co_1.29_Ni_1.72_O_4_ (311). D) HAADF–STEM image and corresponding STEM–EDS elemental mapping of Ru, Mn, Co, Ni, and O, demonstrating uniform spatial distribution of all constituent elements across the catalyst.

TEM images (**Figure** **1**A) showed that the MCN–RuO_2_ material consists of nanoparticles that are relatively uniform and quasispherical in shape. Histogram analysis of particle size distribution (Figure S3, Supporting Information) gave an average diameter of ≈3.2 nm. These features are expected to maximize the electrochemically active surface area (ECSA). Scanning electron microscopy (SEM) images (Figure S4, Supporting Information) agree with these observations and reveal the formation of a rough and porous surface texture, indicative of high surface accessibility.

SAED patterns (Figure 1B) displayed clear diffraction rings characteristic of a polycrystalline structure. The rings were indexed to multiple crystalline phases, including RuO_2_ (110), Co_1.29_Ni_1.72_O_4_ (311), MnO (200), and NiO (200). HRTEM images (Figure 1C) further confirmed these assignments, with lattice spacings of 0.315 and 0.308 nm corresponding to the (110) planes of RuO_2_, 0.219 nm corresponding to MnO (200), 0.208 nm corresponding to NiO (200), and 0.245 nm corresponding to the (311) plane of Co_1.29_Ni_1.72_O_4_. The (311) reflection was selected as representative for the Co_1.29_Ni_1.72_O_4_ spinel because it corresponds to the most intense peak in the reference XRD pattern (PDF 00‐040‐1191), and the d‐spacing measured by SAED (≈0.245 nm) matches the interplanar spacing of this plane, confirming its assignment. The coexistence of these multiple oxide phases provides evidence for the formation of multicomponent oxide system with abundant heterointerfaces. Such interfaces are attractive for tuning the local electronic structure and improving catalytic performance.

High‐angle annular dark field (HAADF) scanning electron transmission microscopy(SETM) imaging (Figure 1D) revealed a uniform distribution of Ru, Mn, Co, Ni, and O across the catalyst. Quantitative elemental analysis by STEM–energy‐dispersive X‐ray spectroscopy (EDS) gave weight percentages of Ru (39.3%), Mn (5.4%), Co (10.5%), Ni (8.6%), and O (35.9%). Bulk composition analysis by MP–AES yielded values of Ru (30.1%), Mn (4.8%), Co (11.0%), and Ni (8.9%). The close agreement confirms consistent multimetal incorporation throughout the catalyst (Table S1, Supporting Information).

**Figure 2:**
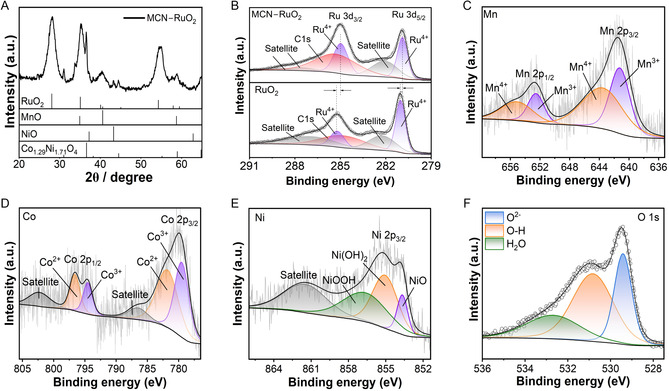
Structural and surface chemistry characterization of MCN–RuO_2_. A) XRD pattern showing RuO_2_ as the dominant phase along with reflections from Mn‐, Ni‐, and Co‐based oxides, indicating a multicomponent heterostructure. B–F) High resolution XPS spectra for (B) Ru 3d, (C) Mn 2p, (D) Co 2p, (E) Ni 2p, and (F) O 1s revealing binding‐energy shifts, mixed‐valent states, and oxygen defect enrichment, consistent with dopant‐induced electronic modulation.

The XRD pattern of MCN–RuO_2_ (**Figure** **2**A) confirms the formation of a multicomponent oxide system. The dominant reflections correspond to the tetragonal RuO_2_ (PDF 00‐043‐1027), establishing it as the primary host lattice. Compared to commercial RuO_2_, the reflections are broadened and slightly shifted to higher angles (Figure S5, Supporting Information), indicating lattice distortion and partial substitution of Ru by Mn, Co, and/or Ni. In addition to the RuO_2_ phase, weaker reflections can be indexed to MnO (PDF 00‐007‐0230), NiO (PDF 00‐047‐1049), and the spinel‐type Co_1.29_Ni_1.72_O_4_ (PDF 00‐040‐1191), consistent with the TEM, HRTEM, and SAED analysis (Figure 1). These secondary oxide domains are likely a consequence of the limited solubility of dopants within the RuO_2_ lattice, resulting in local phase segregation and the formation of rock‐salt and spinel‐type structures. The coexistence of a RuO_2_ matrix with segregated Mn, Co, and Ni oxide phases suggests a dual structural role of the dopants: 1) partial incorporation into the RuO_2_ lattice, which introduces lattice strain and electronic perturbation and 2) stabilization of separate oxide domains, which generate heterointerfaces. Such structural modifications are known to tune the electronic environment, create defect sites, and enrich the diversity of active sites, all of which are expected to synergistically enhance OER activity.^[^ ^26^, ^27^, ^28^ ^]^ These structural features directly reflect the impregnation–annealing protocol. Homogeneous precursor deposition during the impregnation step leads to uniformly distributed nanoscale crystallites after annealing (TEM/SEM, Figure 1A and S4, Supporting Information), while annealing at 450 °C crystallizes the tetragonal RuO_2_ host and drives partial dopant incorporation together with controlled phase segregation into MnO, NiO, and Co_1.29_Ni_1.72_O_4_ domains (SAED and HRTEM, Figure 1B,C; XRD, Figure 2A and S5, Supporting Information).

The survey X‐ray photoelectron spectroscopy (XPS) spectrum of the MCN–RuO_2_ sample (Figure S6, Supporting Information) confirmed the presence of Ru, Mn, Co, Ni, and O, along with a minor C 1s peak originating from adventitious carbon. High‐resolution XPS spectra of the Ru 3d core level (Figure 2B) revealed the characteristic Ru^4+^ doublet, with Ru 3d_5/2_ and Ru 3d_3/2_ peaks centered at ≈280.8 and ≈285 eV, respectively.^[^ ^13^ ^]^ Compared to commercial RuO_2_, MCN–RuO_2_ displayed a slight positive shift in Ru^4+^ binding energy, accompanied by enhanced satellite intensity. These features indicate electron redistribution induced by dopant incorporation, consistent with charge‐transfer interactions between Ru and the Mn, Co, and Ni species.^[^ ^19^ ^]^ Such modifications suggest a perturbed electronic environment for Ru sites, which has been correlated with improved OER activity.

The XPS spectrum of the Mn 2p core level (Figure 2C) displayed Mn 2p_3/2_ and Mn 2p_1/2_ peaks at ≈641.7 and ≈653.5 eV, respectively, consistent with mixed Mn^3+^/Mn^4+^ oxidation states.^[^ ^18^ ^]^ The coexistence of multiple valencies is expected to facilitate redox flexibility and enhance oxygen vacancy formation.^[^ ^29^ ^]^ The Co 2p core‐level XPS spectrum (Figure 2D) exhibited Co 2p_3/2_ and 2p_1/2_ peaks at ≈780.2 and ≈795.3 eV, with prominent satellite features, confirming the presence of both Co^2+^ and Co^3+^ species.^[^ ^30^ ^]^ Relative to cobalt oxides, the observed binding energy shifts suggest strong electronic coupling between Co and the surrounding Ru, Mn, and Ni centers. Similarly, the Ni 2p region (Figure 2E) revealed Ni 2p_3/2_ peaks at 854.1 and 855.8 eV, corresponding to Ni^2+^ and Ni^3+^, together with a characteristic satellite feature at ≈860.5 eV.^[^ ^7^ ^]^ The stabilization of mixed Ni oxidation states is indicative of synergistic metal–metal interactions within the multicomponent system. The O 1s core‐level XPS spectrum (Figure 2F) was deconvoluted into three main contributions at ≈529.5, 531.0, and 533.0 eV, assigned to lattice oxygen (M–O), surface hydroxyl groups (M–OH), and adsorbed water, respectively.^[^ ^31^ ^]^ Together, these XPS results demonstrate that incorporation of Mn, Co, and Ni into the RuO_2_ matrix not only modifies the electronic states of Ru but also stabilizes mixed‐valent dopant species and enriches oxygen defect chemistry. These effects are expected to facilitate charge transfer, optimize adsorption energetics, and ultimately enhance OER catalytic performance.

### 2.2 Electrocatalytic OER Performance in Alkaline Media

**Figure 3:**
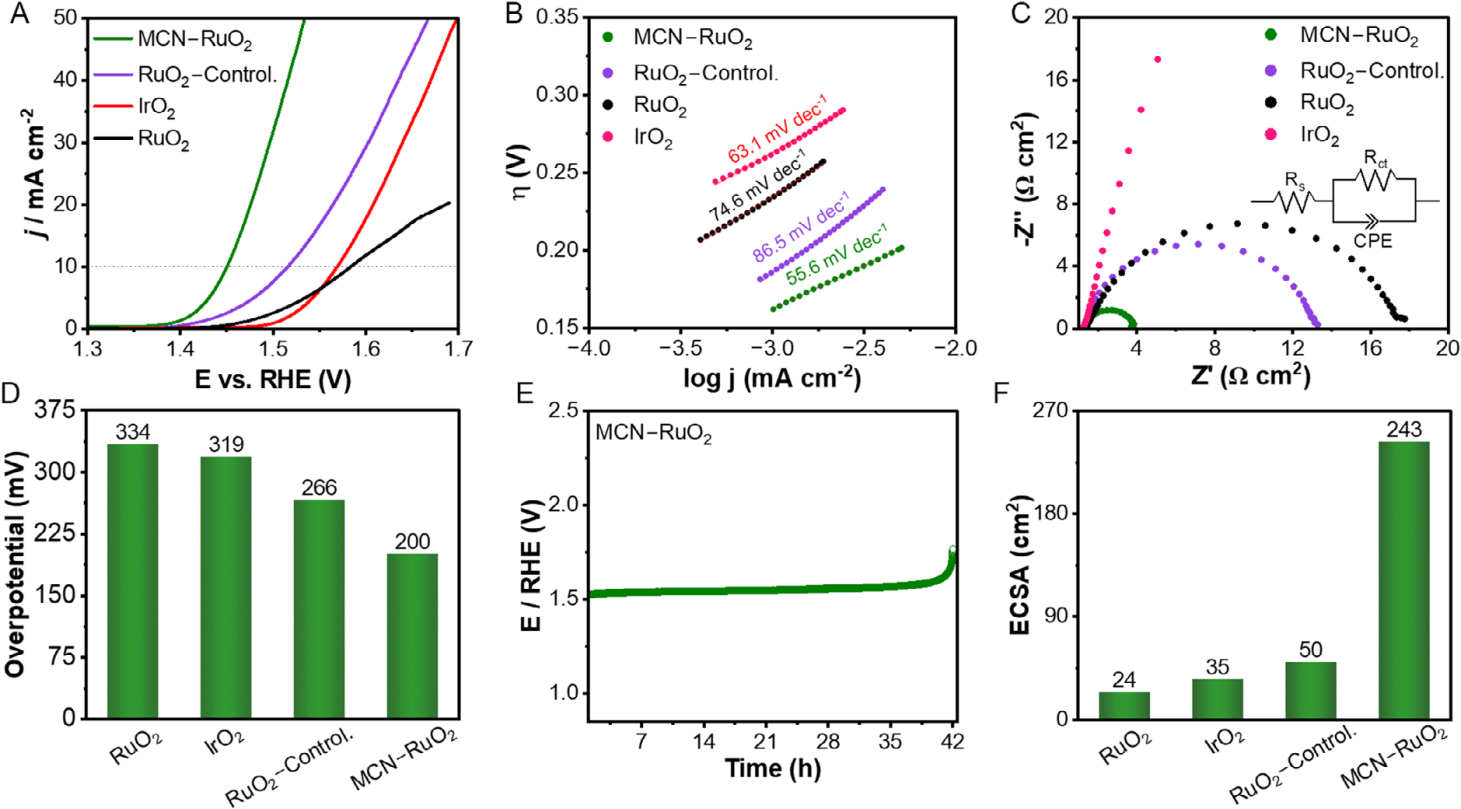
Alkaline OER performance of MCN–RuO_2_ in 1 M KOH. A) *iR*‐corrected polarization curves compared to commercial RuO_2_ and IrO_2_ as well as the control RuO_2_ sample. B) Tafel plots demonstrating faster reaction kinetics for the doped catalyst, reflected in the lowest slope among all samples. C) Nyquist plots from EIS at 1.46 V vs. RHE, indicating reduced charge‐transfer resistance for MCN–RuO_2_. D) Overpotentials at 10 mA cm^−2^, where MCN–RuO_2_ exhibits the lowest value. E) Chronopotentiometry at 10 mA cm^−2^, highlighting the long‐term durability of MCN–RuO_2_. F) ECSA from double‐layer capacitance, showing a higher density of accessible active sites for the doped catalyst.

Having established the structural and electronic modifications introduced by multimetal incorporation, we next evaluated the oxygen evolution performance of MCN–RuO_2_ in 1 M KOH using a standard three‐electrode configuration. To ensure that the choice of MCN**–**RuO_2_ was based on rational optimization rather than arbitrary selection, we systematically benchmarked its OER performance relative to single‐ and binary‐doped RuO_2_ catalysts prepared under identical synthesis and testing conditions (Figure S7, Supporting Information). The linear sweep voltammetry (LSV) curves reveal that while single‐metal doping (Mn, Co, or Ni**–**RuO_2_) improves activity relative to pristine RuO_2_, the gains are modest. For some binary‐doped systems (MnCo, MnNi, and CoNi**–**RuO_2_), further enhancements in performance were detected, highlighting the positive effects of combining two transition metals. Notably, the ternary MCN**–**RuO_2_ catalyst exhibited the lowest onset potential and highest current density among all compositions, clearly outperforming both single‐ and double‐doped counterparts. Thus, we focused on the MCN**–**RuO_2_ for detailed electrochemical testing, mechanistic evaluation, and benchmarking against commercial RuO_2_ and IrO_2_ catalysts as well as a control sample comprised of RuO_2_ prepared by a similar synthesis but in the absence of any dopants (**Figure** **3**A–F).

The LSV curves (Figure 3A) clearly demonstrate the superior activity of MCN–RuO_2_ compared to commercial RuO_2_ and IrO_2_ as well as the RuO_2_ control sample. The optimized catalyst exhibited a low onset potential of 142 mV, significantly lower than that of RuO_2_ (215 mV) and IrO_2_ (242 mV). This substantial activity enhancement is consistent with the lattice distortion, electronic redistribution, and heterointerface formation observed in XRD and XPS analyses, which collectively facilitate more favorable adsorption energetics for OER intermediates.^[^ ^32^ ^]^ The *iR*‐corrected polarization curves are shown in Figure S8, Supporting Information, and further confirm the intrinsic superiority of the doped system. These results highlight that the deliberate integration of Mn, Co, and Ni into the RuO_2_ framework, together with the stabilization of secondary oxide phases, effectively optimizes the active sites and accelerates charge‐transfer kinetics, yielding a highly efficient catalyst under alkaline conditions.

The Tafel slope analysis further highlights the superior kinetics of MCN–RuO_2_ (Figure 3B). The optimized catalyst exhibited a slope of 55.6 mV dec^−1^, which is substantially lower than those of IrO_2_ (63.1 mV dec^−1^) and RuO_2_ (74.6 mV dec^−1^). The reduced slope demonstrates more favorable reaction kinetics and a lower energy barrier for the rate‐determining *O → *OOH step.^[^ ^33^, ^34^ ^]^ Together with the small nanoparticle size and high density of heterointerfaces revealed by TEM and XPS, these factors reduce kinetic barriers and enable more efficient charge transport. The results confirm that the rationally engineered multimetal heterostructure not only lowers the overpotential but also fundamentally improves the intrinsic OER reaction pathway.

Electrochemical impedance spectroscopy (EIS) measurements (Figure 3C) provided further insights into charge‐transfer behavior. The Nyquist plots were fitted using a simplified Randles equivalent circuit, comprising a solution resistance (*R*_s_) in series with a parallel combination of charge‐transfer resistance (*R*_ct_) and a constant phase element, as shown in the inset of Figure 3C. At 1.46 V vs. reversible hydrogen electrode (RHE), MCN–RuO_2_ exhibited the lowest charge‐transfer resistance among all samples (Table S2, Supporting Information), confirming its superior electrical conductivity and interfacial charge transport properties. The enhanced conductivity is attributed to the nanoscale particle size and abundant heterointerfaces, which increase electrochemically accessible surface area while facilitating rapid electron flow. The Tafel slope and EIS analyses clearly demonstrate that the rationally engineered MCN–RuO_2_ heterostructure accelerates OER kinetics through simultaneous optimization of electronic structure and charge‐transfer pathways. This decrease in polarization (charge‐transfer) resistance can be attributed to the combined effects of the optimized electronic structure and enlarged ECSA. DFT calculations (Figure 5) show that multimetal incorporation into RuO_2_ induces pronounced charge redistribution at the heterointerfaces, lowers the work function, and shifts the Ru‐4d states toward the Fermi level, all of which facilitate faster electron transport. At the same time, MCN–RuO_2_ exhibits a substantially higher ECSA than pristine RuO_2_ and IrO_2_ (Figure 3F, S9, and S10, Supporting Information), arising from its ultrafine nanoparticle size and rough, porous morphology (Figure 1A and S4, Supporting Information), which increase the density of accessible active sites and improve electrolyte and substrate contact. Together, these electronic and interfacial/morphological factors synergistically reduce the interfacial charge‐transfer resistance observed in the Nyquist plots.

The overpotentials required to reach 10 mA cm^−2^ (Figure 3D) further emphasize the superior performance of MCN–RuO_2_. The optimized catalyst required only 200 mV, which is substantially lower than that of commercial RuO_2_ (319 mV) and IrO_2_ (334 mV), as well as the control RuO_2_ sample (298 mV). This reduction highlights the effectiveness of multimetal incorporation in lowering the energy barrier for OER and providing a clear advantage over benchmark catalysts. Durability was next assessed by chronopotentiometry at a constant current density of 10 mA cm^−2^ (Figure 3E). MCN–RuO_2_ demonstrated good stability, sustaining nearly constant performance for 42 h before any noticeable degradation. This lifetime surpasses that of commercial RuO_2_ and IrO_2_ as well as the RuO_2_ control sample (Figure S11, Supporting Information) as well as many recently reported non‐Ir‐based catalysts tested on glassy carbon electrodes (Table S3, Supporting Information). The superior stability of MCN–RuO_2_ relative to undoped RuO_2_ is consistent with our DFT results, which show that multimetal doping increases the binding energy of Ru and raises the dissolution potential (Figure S23, Supporting Information, DFT section), thereby suppressing Ru leaching and overoxidation. In addition, the heterogeneous Mn/Co/Ni oxide domains act as redox‐active and mechanically robust “buffer” phases that help to redistribute local strain and charge during operation, mitigating lattice‐oxygen‐mediated degradation pathways and reducing the likelihood of particle detachment. Together, these electronic and structural stabilization effects are in line with the nearly flat chronopotentiometric response, and the preserved nanostructured morphology observed after long‐term operation (Figure 3E and S12, Supporting Information).

Poststability SEM analysis (Figure S12, Supporting Information) further confirmed the robustness of MCN–RuO_2_, revealing that the catalyst retained its nanostructured morphology with no significant changes in particle size, surface texture, or agglomeration after prolonged operation. These results underscore the stabilizing role of Mn, Co, and Ni dopants, which mitigate Ru dissolution and preserve the structural integrity of the active sites under harsh OER conditions.

The ECSA was estimated from the double‐layer capacitance (*C*_dl_) obtained by cyclic voltammetry (Figure S9A–D, Supporting Information). As shown in Figure 3F, MCN–RuO_2_ exhibited the highest *C*_dl_ (Figure S10, Supporting Information) and corresponding ECSA, nearly nine times greater than commercial RuO_2_ and substantially higher than IrO_2_ and the RuO_2_ control. This enhancement reflects the increased density of accessible active sites, consistent with the ultrafine particle size observed by TEM and the defect‐rich surface revealed by XPS. To further assess intrinsic performance, we normalized the OER current density by the mass of noble metal (Ru or Ir) in the catalyst layer (Figure S13, Supporting Information). The resulting mass activities confirm that MCN–RuO_2_ outperforms commercial RuO_2_, IrO_2_, and the undoped RuO_2_ control, demonstrating that the activity gain is not merely due to higher loading but arises from the multimetal‐doped heterostructure design.

Taken together, the electrochemical analyses clearly demonstrate the superior OER performance of MCN–RuO_2_ in alkaline media. The catalyst achieves lower overpotentials and higher current densities than commercial RuO_2_ and IrO_2_, while also exhibiting reduced Tafel slopes and minimized charge‐transfer resistance, indicative of accelerated reaction kinetics and efficient interfacial electron transport. In addition, MCN–RuO_2_ delivers outstanding durability, maintaining stable operation for more than 40 h, and provides the highest ECSA among the tested catalysts, reflecting a high density of accessible active sites. Importantly, the intrinsic activity advantage remains even after ECSA normalization, confirming that the improvements arise from the deliberate structural and electronic modifications introduced by Mn, Co, and Ni incorporation rather than surface area effects alone. These results validate our rational design strategy, where lattice distortion, mixed‐valent dopants, and heterointerface formation collectively optimize activity, stability, and conductivity, positioning MCN–RuO_2_ as a competitive non‐Ir‐based electrocatalyst for alkaline OER. We note that a detailed, quantitative analysis of metal dissolution and surface‐state evolution by inductively coupled plasma mass spectrometry (ICP–MS) and post‐operando XPS would provide additional insight into the antidissolution mechanism and represents an important direction for future work.

In addition to its excellent activity in alkaline media, MCN–RuO_2_ also demonstrated superior OER performance under acidic conditions (0.5 M H_2_SO_4_). The catalyst outperformed commercial RuO_2_ and IrO_2_ in terms of activity, charge‐transfer efficiency, and durability (Figure S14–S18 and Table S4–S5, Supporting Information), sustaining stable operation for extended periods while requiring a lower overpotential to reach benchmark current densities. These results confirm that the synergistic multimetal incorporation strategy is effective across the full pH window relevant to water electrolysis, underscoring the versatility and robustness of MCN–RuO_2_ for practical applications.

### 2.3 Alkaline Exchange Membrane Water Electrolyzer (AEMWE) Performance

**Figure 4:**
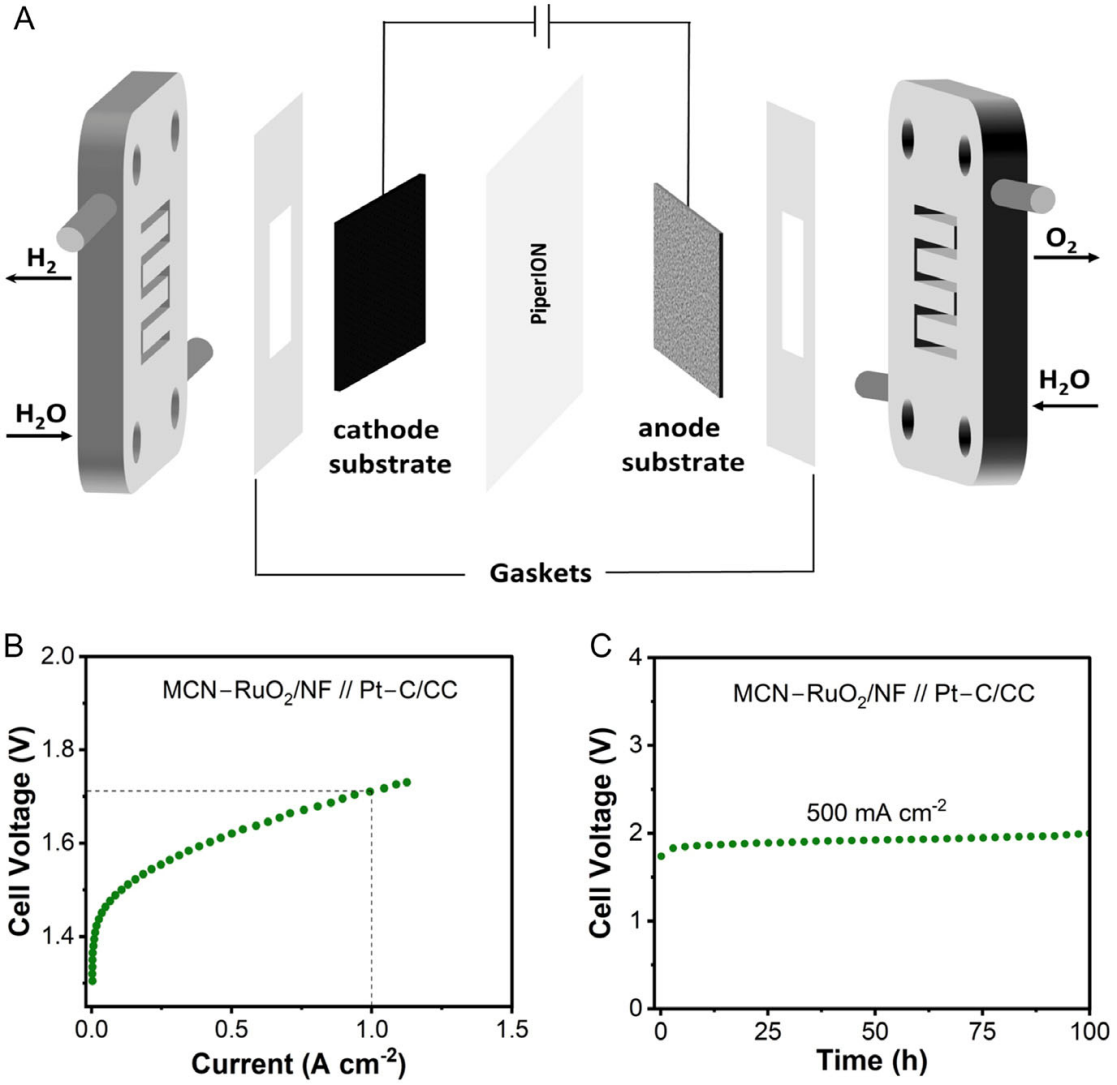
AEMWE performance of MCN–RuO_2_. A) Schematic of the AEMWE single‐cell configuration with catalyst‐coated substrates, bipolar plates, and electrode supports. B) Polarization curve of a single cell using MCN–RuO_2_ as the anode (NF support) and Pt–C as the cathode, achieving 1 A cm^−2^ at 1.71 V. C) Chronopotentiometry at 500 mA cm^−2^ for 100 h, showing excellent stability with minimal voltage degradation. These results highlight the robustness and Ir‐free applicability of MCN–RuO_2_ for device‐level electrolysis.

Encouraged by the high activity and durability of MCN–RuO_2_ in half‐cell tests, we integrated the catalyst into an anion exchange membrane water electrolyzer (AEMWE) to assess its practical performance. AEMWEs are emerging as a promising alternative to proton exchange membrane devices, as they operate in alkaline electrolyte, allow the use of earth‐abundant catalysts and cheaper cell components, and have the potential to combine high efficiency with reduced system cost.^[^ ^35^ ^]^ To assess the applicability of MCN–RuO_2_ under such conditions, we assembled a single‐cell device with an active area of 4.84 cm^2^ using MCN–RuO_2_ as the anode and commercial Pt–C as the cathode (**Figure** **4**A). To evaluate substrate compatibility, both nickel foam (NF) and titanium mesh (TM) were tested as anode supports. In both cases, comparable performance was observed: the NF‐supported catalyst achieved 1 A cm^−2^ at 1.71 V (Figure 4B), while the TM‐supported system required 1.74 V to reach the same current density (Figure S19A,B, Supporting Information). This confirms the versatility of MCN–RuO_2_ across different substrates.

For benchmarking, commercial RuO_2_ and IrO_2_ were evaluated under identical conditions (Figure S19C,D, Supporting Information). MCN–RuO_2_ consistently outperformed these reference catalysts and exceeded the activity of many state‐of‐the‐art‐based anodes reported in the literature (Table S6, Supporting Information). Durability was tested under industrially relevant conditions at 500 mA cm^−2^. The NF‐supported MCN–RuO_2_ catalyst sustained stable operation for 100 h with a low degradation rate of 1.35 mV h^−1^ (Figure 4C). Similar stability was observed with the TM substrate, where the degradation rate was slightly lower (1.06 mV h^−1^, Figure S19B, Supporting Information). These results highlight the robustness, versatility, and practical applicability of MCN–RuO_2_ as a non‐Ir‐based anode catalyst for AEMWE.

### 2.4 DFT Simulations and Mechanistic Insights

To gain atomic‐level understanding of the enhanced OER performance of MCN–RuO_2_, we performed systematic DFT calculations. Guided by experimental observations, structural models were constructed to capture the heterointerfaces formed between RuO_2_ and transition‐metal oxides (TMOs), including RuO_2_/CoNiO_*x*_, RuO_2_/MnO, and RuO_2_/NiO (Figure S20, Supporting Information). Because the experimental MCN–RuO_2_ catalyst is a compositionally complex, disordered quaternary system, we adopted a hierarchical modeling strategy in which simplified RuO_2_/TMO heterointerfaces and monodoped RuO_2_ models are used to isolate the individual contributions of Mn, Co, and Ni, while an explicit multidoped MCN–RuO_2_ supercell (Figure S22, Supporting Information) serves to validate that the key electronic trends carry over to the full multimetal environment. This approach captures the relevant local bonding environments and interfacial charge redistribution in MCN–RuO_2_, while remaining computationally tractable.

We note that the XRD, XPS, and microscopy analyses presented here capture the as‐synthesized state of MCN–RuO_2_. Under alkaline OER conditions, TMOs often undergo surface hydroxylation and partial transformation into (oxy)hydroxide‐like layers. In this context, the RuO_2_/MnO, RuO_2_/NiO, and RuO_2_/CoNiO_*X*_ models used in our DFT study can be viewed as reasonable approximations of RuO_2_ cores interfaced with dynamically formed Mn/Co/Ni (oxy)hydroxide‐like surface domains. The excellent long‐term stability and preserved nanostructured morphology after operation (Figure 3E and S12, Supporting Information) suggest that the underlying RuO_2_–TMO heterointerface remains intact, even if the outermost layers are partially reconstructed during OER.

**Figure 5:**
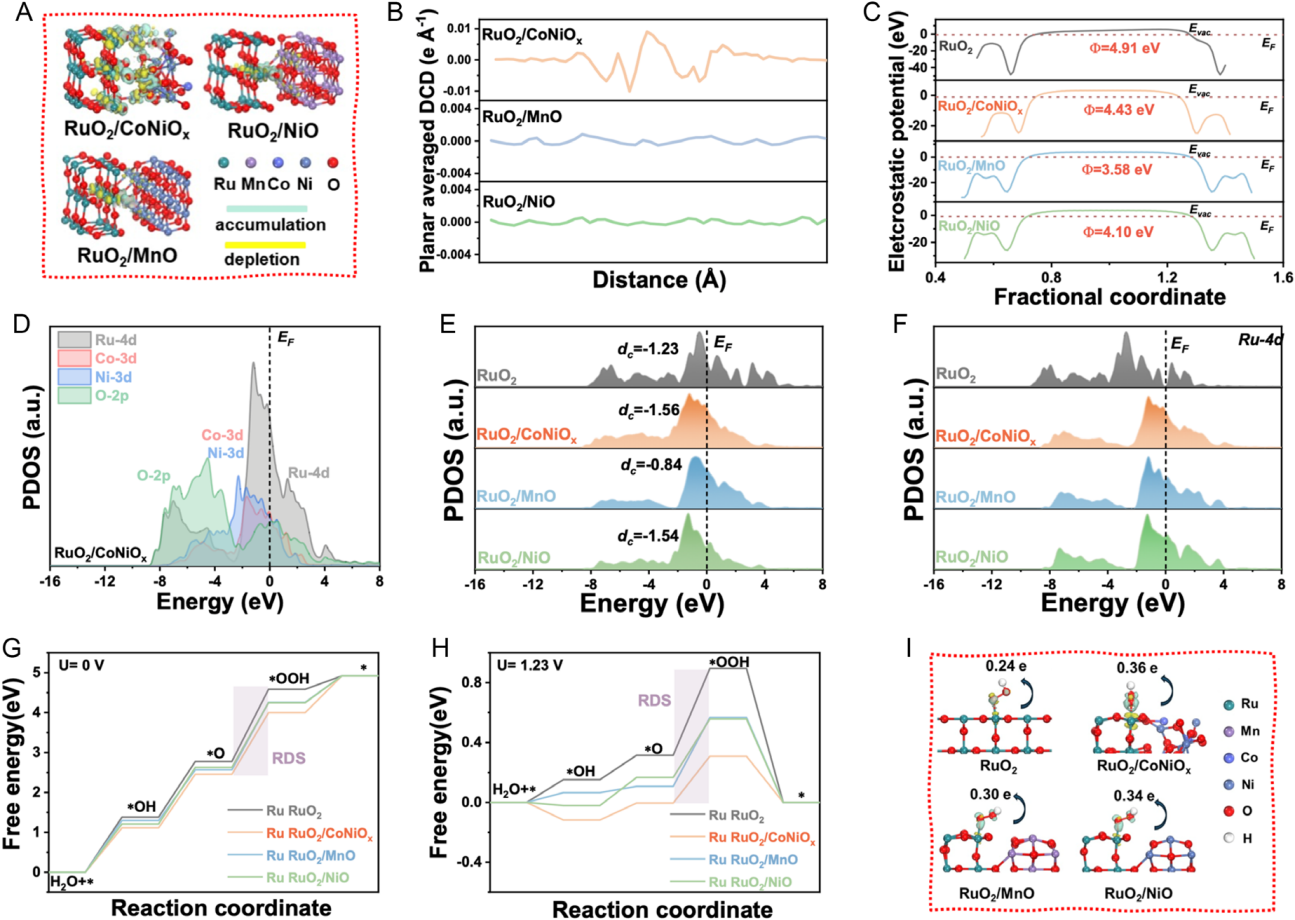
DFT analysis of electronic structure and OER mechanism. A) EDD plots showing charge accumulation (green) and depletion (yellow) across RuO_2_/TMO interfaces. B) Plane‐averaged DCD profiles highlighting enhanced interfacial charge redistribution in RuO_2_/CoNiO_*x*_. C) Electrostatic potential profiles of pristine and composite models, showing reduced work functions relative to RuO_2_. D–F) PDOS analyses demonstrating strong O‐2p and transition‐metal d orbital hybridization and an upshift of Ru‐4d states, particularly in RuO_2_/CoNiO_*x*_. G,H) Free energy diagrams for acidic OER at 0 and 1.23 V, identifying *O → *OOH as the RDS and revealing the lowest theoretical overpotential for RuO_2_/CoNiO_*x*_. I) EDD maps of *OOH adsorption configurations, confirming stronger charge transfer and interfacial stabilization at the RuO_2_/CoNiO_*x*_ interface.

The electron density difference (EDD) analysis (**Figure** **5**A) revealed pronounced charge redistribution in the RuO_2_/CoNiO_x_ system, with distinct regions of electron accumulation and depletion across the interface, compared to the relatively weaker interactions in RuO_2_/MnO and RuO_2_/NiO. This trend was corroborated by the planar‐averaged differential charge density (DCD) profiles (Figure 5B), which show that RuO_2_/CoNiO_*x*_ undergoes stronger electron transfer and interface polarization. In the DCD analysis, the z‐direction is defined along the surface normal, with *z* = 0 set at an interfacial O layer on the RuO_2_ side and the profile taken across the heterointerface toward the secondary oxide. The peaks therefore align with alternating O layers that accumulate charge and metal planes that donate charge, producing a periodic modulation associated with the lattice stacking rather than a random distribution.

Electrostatic potential calculations further indicated that all heterostructures exhibit reduced work functions relative to pristine RuO_2_ (Figure 5C), corresponding to an upshifted Fermi level and enhanced electronic conductivity. These features are expected to facilitate interfacial charge migration during OER, providing a mechanistic rationale for the experimentally observed performance enhancements.^[^ ^36^, ^37^, ^38^ ^]^

The local electronic structures of the different heterointerfaces were further analyzed through partial density of states (PDOS) calculations (Figure 5D–F). In RuO_2_/CoNiO_*x*_, the O‐2p orbitals exhibited strong overlap with the transition‐metal d orbitals, indicating efficient site‐to‐site electron delocalization and charge redistribution across the interface. Similar but weaker hybridization features were observed in RuO_2_/MnO and RuO_2_/NiO (Figure S21, Supporting Information). The Co‐ and Ni‐3d states contributed significantly near the Fermi level, enhancing the system's capacity for electron depletion and facilitating stronger interactions with oxygen intermediates.

Importantly, the heterointerfaces also induced a shift in the d‐band center relative to pristine RuO_2_ (Figure 5E), reflecting modified adsorption properties for OER intermediates. Among the models, RuO_2_/CoNiO_*x*_ exhibited the largest upshift of the Ru‐4d states (Figure 5F), signifying strengthened orbital hybridization and optimized binding of oxygen species. To further link these electronic changes to defect chemistry, we estimated the Ru‐4d band centers from the PDOS relative to the Fermi level (Figure 5E). Compared to pristine RuO_2_, all heterostructure models show a moderate shift of the Ru‐4d band center toward the Fermi level, most pronounced for RuO_2_/CoNiO_X_ and MCN–RuO_2_, indicating increased occupation of antibonding Ru–O states and a concomitant weakening of the Ru–O interaction, which facilitates the formation of oxygen vacancies at the interface. This picture is consistent with the XPS O 1s spectra (Figure 2F), which reveal an enhanced vacancy‐related oxygen component together with mixed‐valent Mn states, suggesting that Mn is particularly effective in promoting vacancy formation, while Co and Ni primarily modulate the local electronic structure and charge transport. These electronic modulations, enabled by the synergistic interactions between Ru and dopant oxides, refine the thermodynamics of intermediate adsorption and provide a direct explanation for the superior catalytic activity observed experimentally.

Motivated by previous studies on RuO_2_‐based OER catalysts in alkaline media and by the fact that the RuO_2_ lattice and oxidation state remain largely preserved under our operating conditions (XRD and XPS, Figure 2A–F and S5, Supporting Information), we considered the conventional adsorbate evolution mechanism, in which OER proceeds via *OH → *O → *OOH on surface Ru sites. Within this framework, the OER reaction pathway was further analyzed by calculating Gibbs free energy profiles for RuO_2_ and the composite models (RuO_2_/CoNiO_*x*_, RuO_2_/MnO, and RuO_2_/NiO) at 0 and 1.23 V versus RHE (Figure 5G,H). At zero potential (Figure 5G), all systems exhibited uphill energy profiles, consistent with the endothermic nature of OER. Across all models, the *O → *OOH transition was identified as the rate‐determining step (RDS), with site‐dependent variations in Δ*G*. Under equilibrium conditions (*U* = 1.23 V, Figure 5H), the RuO_2_/CoNiO_*x*_ interface showed the lowest theoretical overpotential (0.31 V), significantly lower than pristine RuO_2_ (0.58 V), RuO_2_/MnO (0.46 V), and RuO_2_/NiO (0.39 V). This indicates that CoNiO_*x*_ incorporation optimizes the Ru electronic environment, reducing the energy barrier for *OOH formation and facilitating a more favorable, near‐exothermic reaction pathway. Complementary charge density difference analyses of *OOH adsorption (Figure 5I) further confirmed stronger charge transfer and interfacial stabilization at the RuO_2_/CoNiO_*x*_ interface.

In alkaline media, the rate‐determining *O → *OOH step is highly sensitive to the local availability of OH^−^ and to interfacial deprotonation kinetics. Although our DFT models do not explicitly resolve OH^−^ transport or local pH gradients, the pronounced charge redistribution and work‐function lowering observed at the RuO_2_/MnO, RuO_2_/NiO, and RuO_2_/CoNiO_*X*_ interfaces (Figure 5A–F) indicate the formation of strongly polarized regions that can stabilize adsorbed OH^−^ and facilitate proton transfer during *O → *OOH formation. The multimetal heterointerfaces therefore act as electronically and ionically favorable junctions: the basic character of the Mn/Co/Ni oxide domains enhances OH^−^ affinity, while the tuned Ru‐4d/O‐2p hybridization on the RuO_2_ side lowers the barrier for *OOH formation. Together with the high ECSA and porous morphology of MCN–RuO_2_ (Figure 1A, 3F, S4, and S9–S10, Supporting Information), which promote rapid electrolyte access, this provides a qualitative picture of how the heterostructure improves local OH^−^ supply and deprotonation kinetics at the active sites.

To further assess the role of dopants, we modeled monodoped (Mn–RuO_2_, Co–RuO_2_, and Ni–RuO_2_) and multidoped (MCN–RuO_2_) systems (Figure S22, Supporting Information). Binding energy and dissolution potential calculations revealed that multidoping not only reduced the theoretical overpotential (0.36 V for MCN–RuO_2_ vs. higher values for monodoped and pristine RuO_2_) but also enhanced thermodynamic and electrochemical stability, as reflected by more negative binding energies and higher dissolution potentials. These results highlight the synergistic role of Mn, Co, and Ni in simultaneously tuning the adsorption energetics of key intermediates and stabilizing the RuO_2_ lattice, providing a mechanistic basis for the experimentally observed activity and durability enhancements. In particular, our data show that the features identified in the mono‐ and binary‐doped systems are preserved and cooperatively enhanced: charge accumulates at Ru–O–(Mn/Co/Ni) linkages, the Ru‐4d states experience a moderate upshift toward the Fermi level, and O‐2p/transition‐metal d hybridization is strengthened, all of which are consistent with the trends deduced from the simpler heterointerface models. Thus, the triple‐element‐doped MCN–RuO_2_ can be viewed as an integrated realization of the electronic effects rationalized from the mono‐ and dual‐doped cases, rather than a qualitatively different system. We note that, while our analysis focuses on the energetics of the key OER intermediates and the associated electronic‐structure changes, a fully explicit treatment of interfacial water dissociation and OH^−^ transport at the heterointerfaces lies beyond the scope of the present work and will be an interesting topic for future studies.

Taken together, the DFT calculations provide a coherent explanation for the experimentally observed performance of MCN–RuO_2_. The heterointerfaces created by multimetal incorporation promote charge redistribution, reduce the work function, and enhance orbital hybridization, thereby optimizing the adsorption thermodynamics of key OER intermediates. Among the models, RuO_2_/CoNiO_*x*_ exhibited the most favorable electronic structure and the lowest theoretical overpotential, consistent with the superior activity measured in half‐cell and device‐level experiments. Furthermore, stability analyses revealed that multidoping strengthens the Ru–O framework against dissolution, rationalizing the extended durability observed in both alkaline and acidic media. These results confirm that the synergistic incorporation of Mn, Co, and Ni not only accelerates OER kinetics but also stabilizes the catalyst under harsh electrochemical conditions, underscoring the effectiveness of our rational design strategy.

## 3 Conclusions

In summary, we have developed a MCN–RuO_2_‐heterostructured catalyst that leverages multimetal doping and phase‐segregated oxide formation to overcome the activity–stability trade‐off in OER electrocatalysis. Structural characterization confirmed the coexistence of a RuO_2_ matrix with MnO and spinel‐type CoNiO_x_ domains, creating defect‐rich interfaces that modulate the electronic structure. Electrochemical testing demonstrated that MCN–RuO_2_ outperforms commercial RuO_2_ and IrO_2_ benchmarks, achieving low overpotentials, favorable kinetics, and excellent stability. When deployed in an AEMWE, the catalyst sustained 100 h of continuous operation at 500 mA cm^−2^ with minimal degradation, highlighting its robustness under device‐level conditions. Complementary DFT simulations revealed that dopant incorporation drives charge redistribution, reduces the work function, and shifts the Ru‐4d band center, thereby lowering the energy barrier of the RDS and enhancing structural stability. These theoretical insights align with experimental results, establishing a clear structure–function relationship. Overall, MCN–RuO_2_ provides a versatile platform effective across both alkaline and acidic electrolytes, positioning it as a strong candidate for Ir‐free electrolyzer technologies. More broadly, this work establishes a blueprint for the rational design of multimetal‐doped heterostructures as next‐generation OER catalysts, bridging fundamental understanding with scalable solutions for sustainable hydrogen production.

## 4 Experimental Section

4.1

4.1.1

##### Materials

Acetone (CH_3_COCH_3_, 99%, Honeywell), isopropanol (C_3_H_8_O, HPLC grade, 99.9%, Sigma– Aldrich), nitric acid (HNO_3_, 65.0–67.0%, Sigma–Aldrich), hydrochloric acid (HCl, 37%, Sigma–Aldrich), sulfuric acid (H_2_SO_4_, ACS reagent, 98% Honeywell), ruthenium chloride hydrate (RuCl_3_.nH_2_O, Merck), cobalt chloride hexahydrate (CoCl_2_.6H_2_O, Merck), manganese dichloride (MnCl_2_.4H_2_O, Merck), anhydrous nickel chloride, NiCl_2_ (Merck), and carbon vulcan XC‐72 (Fuel Cell Store) were used as received. Absolute ethanol was purchased from Thermo Scientific and used without further purification. Deionized (DI) water (18.2 MΩcm, Milli‐Q purification system) was used throughout the experiments.

##### Physical Characterizations

SEM images were obtained using a Hitachi S–4800 field emission microscope, operating at an accelerating voltage of 30 kV and interfaced with EDS. SEM samples were prepared by dispersing the nanoparticle suspension in ethanol with an ultrasonic bath and drop casting onto a Si wafer, followed by drying under ambient conditions before imaging. TEM images were obtained on a Jeol JEM‐1400 TEM, operating at an accelerating voltage of 120 kV. HRTEM (FEI Titan Cubed 60–300) was used to resolve the structural details with an operating voltage of 300 kV. Furthermore, the HAADF and composition examinations were conducted on a Talos F200X transmission electron microscope with an EDS detector. (S)TEM samples were prepared by dispersing the nanoparticle suspension in ethanol with an ultrasonic bath and drop casting onto carbon‐coated copper grids. Microwave plasma atomic emission spectroscopy (AES) (Agilent Technologies MP–AES 4100 spectrometer) was used to quantify the metal content. All solutions were prepared using 0.5 M HCl solution. Standard metal solutions 1, 2, 5, 10, and 20 ppm for equipment calibration were prepared from standard 1000 ppm solution of each metal. The catalyst analysis solutions were prepared by dissolving 1 mg in 4 mL of aqua regia. After evaporation part of the liquid, the catalyst was dispersed in 10 mL of 0.5M HCl solution.

XPS was performed using a PREVAC spectrometer with a monochromatized Al Kα anode (1486.7 eV) under ultrahigh vacuum (10^−10^ mbar). The survey spectra were measured with 200 eV pass energy, and high‐resolution spectra were measured with 100 eV pass energy. Casa XPS software was used for data interpretation. To investigate the crystal structure, XRD was performed using a PANalytical X’Pert PRO diffractometer equipped with a Cu Kα1 X‐ray source, working at 45 kV and beam current fixed at 40 mA. X‐ray diffractograms were obtained with a scanning rate of 0.2° min^−1^ and a step size of 0.02°. The sample was measured in a 2*θ* range from 20° to 65°.

##### Synthesis of MCN–RuO_*2*_ *Catalysts*

In a typical synthesis, 0.15 mmol each of RuCl_3_·3H_2_O, CoCl_2_·6H_2_O, and anhydrous NiCl_2_, together with 0.075 mmol of MnCl_2_·4H_2_O, was dissolved in 40 mL of 1 M HCl solution and sonicated for 2 h. This acid etching step selectively removes unstable or weakly bound surface species and exposes catalytically accessible oxide interfaces, as corroborated by the improved OER performance discussed in the Results and Discussion. Carbon vulcan (80 mg) was then added, and the suspension was stirred for 18 h at room temperature to ensure uniform distribution. The metal precursors were converted to their corresponding oxides by annealing at 450 °C for 3 h in air using a muffle furnace (Nabertherm, Germany) with a controlled heating rate of 2 °C min^−1^. After annealing, the material was dispersed in 20 mL of 1 M HCl solution and stirred for 12 h. After acid etching, the samples were washed with DI water by centrifugation at 7400 rpm for 10 min per cycle, repeated four times. The final product was then dried in an oven at 60 °C. A similar synthesis procedure without any doping agents was performed to prepare a RuO_2_ control sample. Also, control samples based on single doping with Co, Ni, or Mn and binary doping using combinations of Co and Ni, Co and Mn, and Ni and Mn were also prepared by a similar procedure employing the corresponding metal precursors.

##### Electrode Preparation and Electrochemical Measurements

Electrochemical measurements were performed on an Autolab PGSTAT128N potentiostat in a standard three‐electrode configuration. An L‐shaped glassy carbon electrode (0.1257 cm^2^) was used as the working electrode (WE), a Pt wire as the counter electrode, and an RHE as the reference electrode. Prior to the electrochemical measurements using the three‐electrode setup, the WE (L‐shaped glassy carbon) was cleaned DI water to remove any surface contaminants. It was then polished on a polishing pad using a 0.05 μm alumina suspension under gentle pressure, moving the electrode in a figure‐eight pattern to ensure a smooth and uniform surface. After polishing, the electrode was thoroughly rinsed with DI water and ultrasonicated in isopropyl alcohol for 30 s. Finally, the electrode was dried at room temperature. Electrolytes were 1 M KOH and 0.5 M H_2_SO_4_. Catalyst inks were prepared by dispersing 5 mg of catalyst in 1 mL of a mixture of Nafion, ethanol, and water (0.1:7.0:2.9 v/v/v), followed by sonication for 60 min to form a homogeneous suspension. Aliquots (6.28 μL, corresponding to a loading of 250 μg cm^−2^) were drop‐cast onto glassy carbon electrodes and dried at 45–50 °C. Prior to measurements, electrodes were cycled between 0.92 and 0.97 V vs. RHE at 50 mV s^−1^ until stable curves were obtained, ensuring a steady surface state. No additional quiet time or resting period was applied before these activation cycles. Polarization curves were then recorded from 1.3 to1.8 V vs. RHE at a scan rate of 5 mV s^−1^. Ohmic drop compensation (85%) was applied using the solution resistance (*R*_s_) obtained from EIS measurements at open‐circuit potential (OCP) to correct for iR losses and ensure accurate evaluation of the intrinsic catalytic activity. Overpotentials (*η*) at a given current density were obtained from the iR‐corrected polarization curves as *η* = *E* − 1.23 V, where *E* is the potential (vs. RHE) required to reach the target current (e.g., 10 mA cm^−2^).

##### AEMWE Measurements

AEMWE measurements were performed using a catalyst‐coated substrate configuration. A PiperION‐A60‐HCO_3_ membrane (Versogen, 60 μm thickness) was employed as the anion exchange membrane, NF and/or TM as the anode substrate, and carbon cloth (CC) as the cathode substrate. Commercial Pt–C (40 wt% Pt, Fuel Cell Store) served as the cathode catalyst, while MCN–RuO_2_ was used as the anode catalyst. Prior to testing, the PiperION membrane was immersed in freshly prepared 1 M KOH for 72 h to fully convert the bicarbonate to the hydroxide form. The TM substrate was cleaned by ultrasonication in 3 M HCl for 15 min, followed by sequential washing with water, acetone, and ethanol. The NF and CC substrates were sequentially washed with acetone and water. 7.26 mg of the MCN–RuO_2_ catalyst was dispersed in a mixture of 2 mL DI water, 2 mL absolute ethanol, and 13.83 μL of 20 wt% Nafion solution to form the catalyst ink. The ink was ultrasonicated for 45 min to achieve a uniform dispersion. The Nafion ionomer loading was 20 wt% relative to the catalyst in both anode and cathode inks. The resulting suspension was then sprayed onto TM or NF substrate using an airbrush at a flow rate of 0.2 mL min^−1^. The coated substrates were dried in a microwave oven at 50 °C for 2–3 h prior to assembly in the electrolyzer. The anode ink was uniformly sprayed onto the cleaned TM, achieving a catalyst loading of 1.5 mg cm^−2^, while the cathode was prepared with a Pt loading of 0.6 mg cm^−2^. During cell assembly, a 0.2 mm polytetrafluoroethylene (PTFE) gasket was used, and the cell was tightened to a torque of 4.0 N m. Electrochemical measurements were carried out at ambient pressure in 1 M KOH electrolyte. Polarization (i‐V) curves were recorded at 60 °C, while durability tests were conducted at 40 °C, with 1 M KOH supplied to both anode and cathode compartments at a flow rate of 20 mL min^−1^. To stabilize the system, 50 consecutive i‐V cycles were performed at a scan rate of 10 mV s^−1^. For AEM catalyst activity and durability evaluation, an *iR*‐compensation of 85% was applied. EIS was conducted with an amplitude of 10 mV over a frequency range of 0.1 Hz–100 kHz at OCP.

##### Theoretical Calculations

DFT calculations were performed using the CASTEP module in Materials Studio.^[^ ^39^ ^]^ Exchange–correlation interactions were treated with the generalized gradient approximation using the Perdew–Burke–Ernzerhof functional.^[^ ^40^ ^]^ Valence–core interactions were described using on‐the‐fly generated ultrasoft pseudopotentials. The valence electron configurations used in the ultrasoft pseudopotentials were Ru (4d^7^5s^1^), O (2s^2^2p^4^), Mn (3d^5^4s^2^), Co (3d^7^4s^2^), and Ni (3d^8^4s^2^). A plane‐wave cutoff energy of 420 eV was applied. A cutoff energy of 420 eV was selected based on convergence tests showing negligible changes in total energy (<1.5 eV) at higher cutoff values. Grimme's D2 scheme was employed to account for van der Waals interactions. The Grimme D2 dispersion correction was applied to all atom pairs in the models (Ru, O, Mn, Co, and Ni), and all doped systems were treated using spin‐polarized calculations. The dispersion settings were kept consistent for clean slabs and adsorbed states to avoid cancellation errors. Spin polarization was included for all Mn/Co/Ni‐containing models; local magnetic moments were initialized on the dopant atoms and allowed to relax self‐consistently to their ground‐state values. These settings ensure a physically accurate and fully converged representation of the electronic structure without requiring additional computations. Geometry optimization was performed using the Broyden–Fletcher–Goldfarb–Shannon algorithm with medium k‐point sampling.^[^ ^41^ ^]^ Brillouin‐zone integrations were performed using a (1 × 1 × 1) Monkhorst–Pack k‐point mesh, which is appropriate for the large lateral dimensions of the heterostructure supercells, where denser sampling provides no additional accuracy. Convergence criteria were set to 5 × 10^−5^ eV/atom (energy), 0.001 eV/Å (force), and 0.005 Å (displacement). The self‐consistent field (SCF) cycle was converged to an electronic energy threshold of 1 × 10^−5^ eV per atom prior to each ionic step. We note that our CASTEP calculations employed Pulay (density) mixing with a maximum of 300 SCF iterations and convergence evaluated over a 3‐step window. These settings provide stable and well‐converged solutions, and no further adjustments were necessary. A vacuum spacing of 20 Å was introduced along the z‐axis to avoid interactions between periodic images.

## Supporting Information

Supporting Information is available from the Wiley Online Library or from the author.

## Conflict of Interest

The authors declare no conflict of interest.

## Supporting information

Supplementary Material

## Data Availability

The data that support the findings of this study are available in the Supporting Information of this article.

## References

[1] J. Song , C. Wei , Z. F. Huang , C. Liu , L. Zeng , X. Wang , Z. J. Xu , Chem. Soc. Rev. 2020, 49, 2196. 32133479 10.1039/c9cs00607a

[2] Y. Zhao , D. P. Adiyeri Saseendran , C. Huang , C. A. Triana , W. R. Marks , H. Chen , H. Zhao , G. R. Patzke , Chem. Rev. 2023, 123, 6257. 36944098 10.1021/acs.chemrev.2c00515

[3] X. Ping , Y. Liu , L. Zheng , Y. Song , L. Guo , S. Chen , Z. Wei , Nat. Commun. 2024, 15, 1. 38509091 10.1038/s41467-024-46815-6 PMC10954744

[4] M. Muhyuddin , C. Santoro , L. Osmieri , V. C. A. Ficca , A. Friedman , K. Yassin , G. Pagot , E. Negro , A. Konovalova , G. Lindquist , L. Twight , M. Kwak , E. Berretti , V. Di Noto , F. Jaouen , L. Elbaz , D. R. Dekel , P. Mustarelli , S. W. Boettcher , A. Lavacchi , P. Atanassov , Chem. Rev. 2025, 125, 6906. 40748968 10.1021/acs.chemrev.4c00466 PMC12355722

[5] N. Du , C. Roy , R. Peach , M. Turnbull , S. Thiele , C. Bock , Chem. Rev. 2022, 122, 11830. 35442645 10.1021/acs.chemrev.1c00854 PMC9284563

[6] S. Li , T. Liu , W. Zhang , M. Wang , H. Zhang , C. Qin , L. Zhang , Y. Chen , S. Jiang , D. Liu , X. Liu , H. Wang , Q. Luo , T. Ding , T. Yao , Nat. Commun. 2024, 15, 1. 38649713 10.1038/s41467-024-47736-0 PMC11035637

[7] Z. Y. Wu , F. Y. Chen , B. Li , S. W. Yu , Y. Z. Finfrock , D. M. Meira , Q. Q. Yan , P. Zhu , M. X. Chen , T. W. Song , Z. Yin , H. W. Liang , S. Zhang , G. Wang , H. Wang , Nat. Mater. 2023, 22, 100. 36266572 10.1038/s41563-022-01380-5

[8] Y. Liu , Y. Wang , H. Li , M. G. Kim , Z. Duan , K. Talat , J. Y. Lee , M. Wu , H. Lee , Nat. Commun. 2025, 16, 1. 39962051 10.1038/s41467-025-56638-8 PMC11832934

[9] J. Zhang , X. Fu , S. Kwon , K. Chen , X. Liu , J. Yang , H. Sun , Y. Wang , T. Uchiyama , Y. Uchimoto , S. Li , Y. Li , X. Fan , G. Chen , F. Xia , J. Wu , Y. Li , Q. Yue , L. Qiao , D. Su , H. Zhou , W. A. Goddard , Y. Kang , Science 2025, 387, 48. 39745949 10.1126/science.ado9938

[10] P. Li , S. Wang , H. Huang , L. Wen , J. Cai , Y. Peng , Z. Zou , X. Wang , X. Fang , L. Fang , X. Wang , Z. Xie , S. Xie , Nano Energy 2025, 139, 110960.

[11] G. Chen , R. Lu , C. Ma , X. Zhang , Z. Wang , Y. Xiong , Y. Han , Angew. Chem. – Int. Ed. 2024, 63, e202411603. 10.1002/anie.202411603 39231800

[12] H. Liu , Z. Zhang , J. Fang , M. Li , M. G. Sendeku , X. Wang , H. Wu , Y. Li , J. Ge , Z. Zhuang , D. Zhou , Y. Kuang , X. Sun , Joule 2023, 7, 558.

[13] Z. Shi , J. Li , Y. Wang , S. Liu , J. Zhu , J. Yang , X. Wang , J. Ni , Z. Jiang , L. Zhang , Y. Wang , C. Liu , W. Xing , J. Ge , Nat. Commun. 2023, 14, 1. 36792586 10.1038/s41467-023-36380-9 PMC9932065

[14] M. Plevová , J. Hnát , K. Bouzek , J. Power Sources 2021, 507, 230072.

[15] C. Rong , K. Dastafkan , Y. Wang , C. Zhao , Adv. Mater. 2023, 35, 2211884. 10.1002/adma.202211884 37549889

[16] X. Wu , Q. Yan , H. Wang , D. Wu , H. Zhou , H. Li , S. Yang , T. Ma , H. Zhang , X. Wu , Q. Yan , H. Wang , D. Wu , H. Zhou , S. Yang , H. Zhang , H. Li , T. Ma , Adv. Funct. Mater. 2024, 34, 2404535.

[17] T. Liu , L. Wang , B. Chen , H. Liu , S. Wang , Y. Feng , J. Zhang , Y. Yin , M. D. Guiver , Angew. Chem. Int. Ed. 2025, 64, e202421869. 10.1002/anie.202421869 39810745

[18] S. Chen , H. Huang , P. Jiang , K. Yang , J. Diao , S. Gong , S. Liu , M. Huang , H. Wang , Q. Chen , ACS Catal. 2019, 10, 1152.

[19] J. Wang , Y. Ji , R. Yin , Y. Li , Q. Shao , X. Huang , J. Mater. Chem. A Mater. 2019, 7, 6411.

[20] J. Huang , A. H. Clark , N. Hales , K. Crossley , J. Guehl , R. Skoupy , T. J. Schmidt , E. Fabbri , Nat. Chem. 2025, 17, 856. 40155757 10.1038/s41557-025-01784-1 PMC12141032

[21] W. Yang , Z. Wang , J. Zhang , L. Jia , J. Li , X. Chen , X. Liu , H. Zhang , J. Lin , M. Zhao , Q. Chen , Angew. Chem. Int. Ed. 2025, 64, e202509768. 10.1002/anie.202509768 40418564

[22] J. Li , M. Yao , Z. Yuan , J. Ma , S. Geng , F. Liu , Chem. Eng. J. 2024, 496, 153921.

[23] W. Zheng , Y. Zhao , K. Jiang , F. Xie , L. Meng , S. Gao , J. Li , J. Lan , M. Luo , L. Liu , Y. Tan , Nat. Commun. 2025, 16, 1. 40691160 10.1038/s41467-025-62036-x PMC12279930

[24] F. Qian , D. Cao , S. Chen , Y. Yuan , K. Chen , P. J. Chimtali , H. Liu , W. Jiang , B. Sheng , L. Yi , J. Huang , C. Hu , H. Lei , X. Wu , Z. Wen , Q. Chen , L. Song , Nat. Commun. 2025, 16, 1. 40715041 10.1038/s41467-025-61763-5 PMC12297328

[25] W. Li , D. Chen , Z. Lou , H. Yuan , X. Fu , H. Y. Lin , M. Lin , Y. Hou , H. Qi , P. F. Liu , H. G. Yang , H. Wang , J. Am. Chem. Soc. 2025, 147, 10446. 40018804 10.1021/jacs.4c18300

[26] T. Wang , Z. Li , H. Jang , M. G. Kim , Q. Qin , X. Liu , ACS Sustain. Chem. Eng. 2023, 11, 5155.

[27] B. Yuan , Q. Dang , H. Liu , M. G. Sendeku , J. Peng , Y. Fan , L. Cai , A. Cao , S. Chen , H. Li , Y. Kuang , F. Wang , X. Sun , Nat. Commun. 2025, 16, 1. 40379743 10.1038/s41467-025-59710-5 PMC12084596

[28] Q. Dang , Z. Shang , F. Wang , X. Sun , H. Li , J. Phys. Chem. C 2025, 128, 17091.

[29] Y. Wang , L. Chen , H. Cao , Z. Chi , C. Chen , X. Duan , Y. Xie , F. Qi , W. Song , J. Liu , S. Wang , Appl. Catal. B 2019, 245, 546.

[30] R. Fantin , A. Van Roekeghem , A. Benayad , Surf. Interface Anal. 2023, 55, 489.

[31] H. Over , Chem. Rev. 2012, 112, 3356. 22423981 10.1021/cr200247n

[32] M. Retuerto , L. Pascual , O. Piqué , P. Kayser , M. A. Salam , M. Mokhtar , J. A. Alonso , M. Peña , F. Calle‐Vallejo , S. Rojas , J. Mater. Chem. A Mater. 2021, 9, 2980.

[33] W. T. Hong , M. Risch , K. A. Stoerzinger , A. Grimaud , J. Suntivich , Y. Shao‐Horn , Energy Environ. Sci. 2015, 8, 1404.

[34] B. Zhang , X. Zheng , O. Voznyy , R. Comin , M. Bajdich , M. García‐Melchor , L. Han , J. Xu , M. Liu , L. Zheng , F. P. G. De Arquer , C. T. Dinh , F. Fan , M. Yuan , E. Yassitepe , N. Chen , T. Regier , P. Liu , Y. Li , P. De Luna , A. Janmohamed , H. L. Xin , H. Yang , A. Vojvodic , E. H. Sargent , Science 2016, 352, 333. 27013427 10.1126/science.aaf1525

[35] G. Sriram , K. Dhanabalan , K. V. Ajeya , K. Aruchamy , Y. C. Ching , T. H. Oh , H. Y. Jung , M. Kurkuri , J. Mater. Chem. A Mater. 2023, 11, 20886.

[36] K. Du , L. Zhang , J. Shan , J. Guo , J. Mao , C. C. Yang , C. H. Wang , Z. Hu , T. Ling , Nat. Commun. 2022, 13, 1. 36114207 10.1038/s41467-022-33150-x PMC9481627

[37] Y. Zhao , Y. Long , W. Liu , Z. Han , Y. Cui , Z. Li , W. Wang , Z. Duan , X. Fu , J. Mater. Chem. A Mater. 2025, 13, 8474.

[38] Y. Qin , T. Yu , S. Deng , X. Y. Zhou , D. Lin , Q. Zhang , Z. Jin , D. Zhang , Y. B. He , H. J. Qiu , L. He , F. Kang , K. Li , T. Y. Zhang , Nat. Commun. 2022, 13, 1. 35778401 10.1038/s41467-022-31468-0 PMC9249734

[39] S. J. Clark , M. D. Segall , C. J. Pickard , P. J. Hasnip , M. I. J. Probert , K. Refson , M. C. Payne , Zeitschrift fur Kristallographie 2005, 220, 567.

[40] M. Ernzerhof , G. E. Scuseria , J. Chem. Phys. 1999, 110, 5029.

[41] S. Wang , H. Yan , W. Huo , A. Davydok , M. Zając , J. Stępień , H. Feng , Z. Xie , J. K. Shang , P. H. C. Camargo , J. Jiang , F. Fang , Appl. Catal. B: Environ. Energy 2025, 363, 124791.

