# Supplementary material for: Multimetal Doping and Heterostructure Engineering of RuO2 for Durable and Efficient Oxygen Evolution
Source: Small Sci. 2025 Dec 24;6(2):e202500546. doi: 10.1002/smsc.202500546 (PMC12910629; doi:10.1002/smsc.202500546)
Supplement: Supplementary file 1 — Supplementary Material [file SMSC-6-e202500546-s001.pdf]

Supporting Information for

Multi-Metal Doping and Heterostructure Engineering of RuO_2 for Durable and Efficient Oxygen Evolution

Md Mofakkharulhashan, Shiqi Wang, Hugo L. S. Santos, Mykhailo Chundak, Mikko Ritala, and Pedro H. C. Camargo*

[1]*University of Helsinki, Department of Chemistry, A.I. Virtasen aukio 1, Helsinki, Finland*

**Corresponding author. Email: pedro.camargo@helsinki.fi*

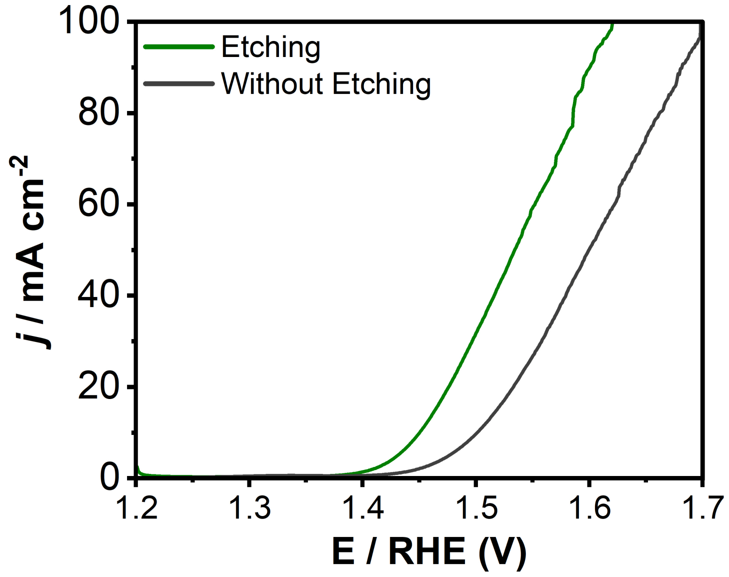

Figure S1. Effect of acid etching on the OER performance of MCN–RuO_2. Linear sweep voltammetry (LSV) curves in 1 M KOH comparing etched and non-etched samples. The etched catalyst exhibits a lower onset potential and higher current density, demonstrating that selective removal of unstable surface species during the etching step is critical for activating the catalyst and exposing accessible active sites.

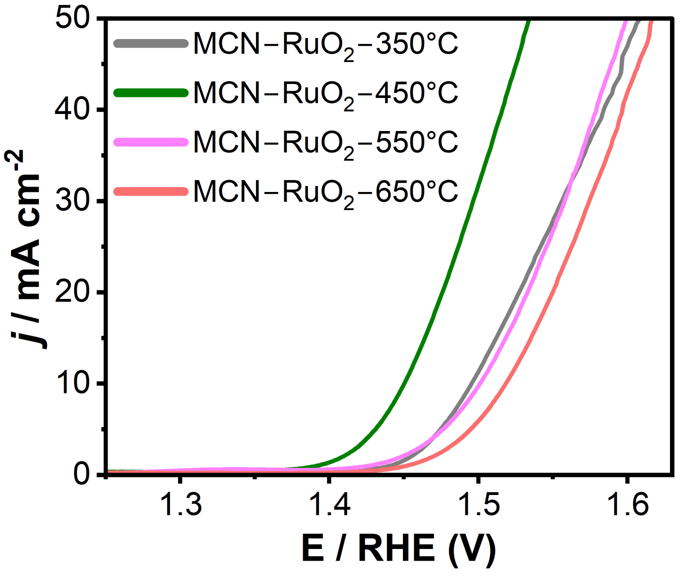

Figure S2. Linear sweep voltammetry (LSV) curves of MCN–RuO_2 catalysts annealed at 350, 450, 550, and 650 °C in air. The sample treated at 450 °C exhibits the lowest onset potential and highest current density, indicating that moderate calcination promotes highly dispersed oxide phases while avoiding excessive particle growth or phase segregation, thereby optimizing OER performance.

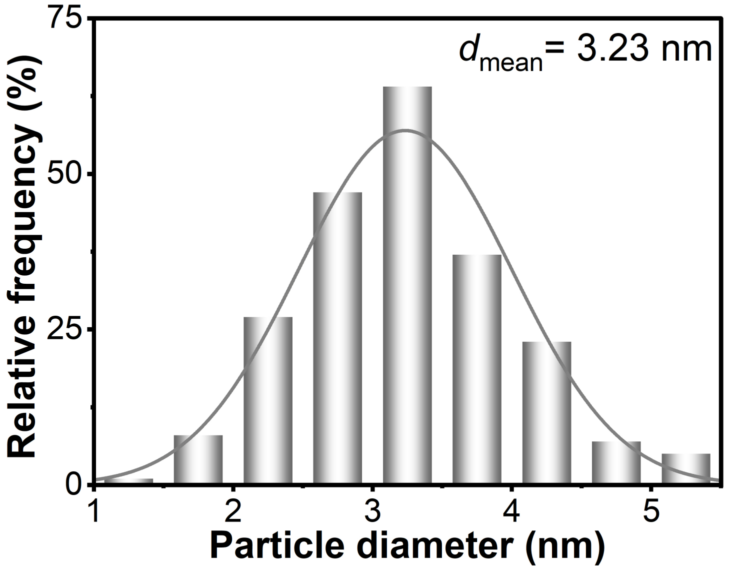

Figure S3. Particle size distribution of MCN–RuO_2 obtained from TEM analysis. The histogram reveals an average particle diameter of ~3.2 nm, confirming the formation of ultrafine nanoparticles that are expected to maximize electrochemically active surface area during OER.

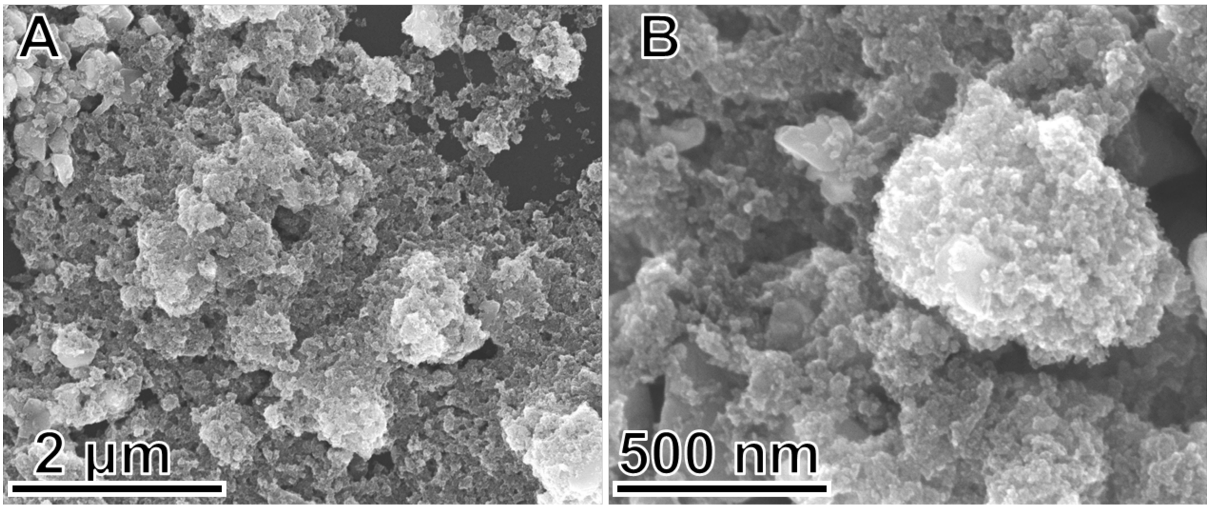

Figure S4. SEM images of MCN–RuO_2. The catalyst exhibits irregularly shaped nanocrystals with aggregated clusters and a porous surface texture, features that are expected to provide high surface accessibility for electrocatalysis.

Table S1. Surface and bulk elemental compositions of MCN–RuO_2 as determined by SEM–EDS and MP–AES, showing consistent dopant incorporation throughout the catalyst.

MCN–RuO_2	SEM-EDX (weight %)	MP-AES (weight %)
**Ru**	39.3 ± 0.3	30.1 ± 0.3
**Mn**	5.4 ± 0.4	4.8 ± 0.3
**Co**	10.5 ± 0.5	11.0 ± 0.2
**Ni**	8.5 ± 0.4	8.9 ± 0.4
**O**	35.9 ± 0.3	-

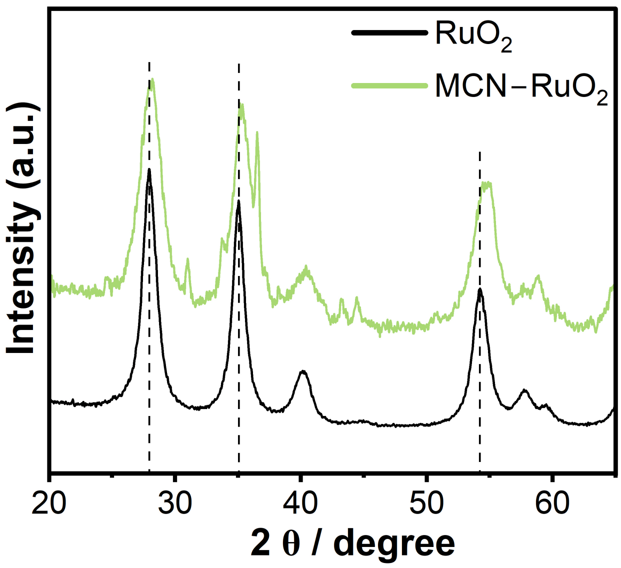

Figure S5. X-ray diffraction (XRD) patterns of MCN–RuO_2 compared with commercial RuO_2. Peak broadening and slight shifts in the synthesized sample indicate lattice distortion and partial dopant incorporation into the RuO_2 matrix.

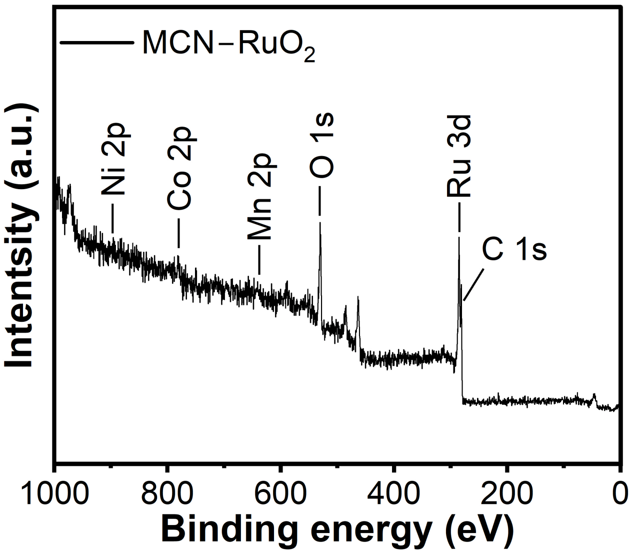

Figure S6. XPS survey spectrum of MCN–RuO_2. Distinct signals for Ru, Mn, Co, Ni, and O confirm successful multi-metal incorporation, while a minor C 1s peak originates from adventitious carbon.

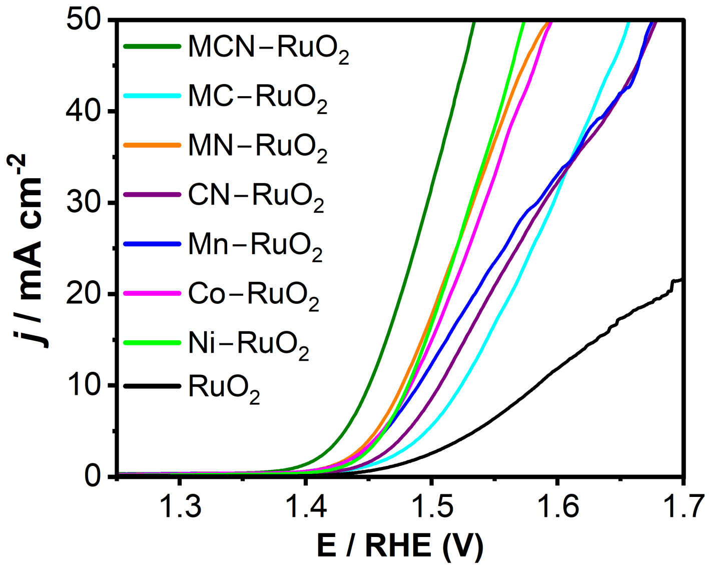

Figure S7. Composition optimization of multi-metal doped RuO_2 catalysts. LSV curves in 1 M KOH comparing single-metal (Mn–, Co–, Ni–RuO_2), binary-metal MnCo (MC–RuO_2), MnNi (MN–RuO_2), CoNi (CN–RuO_2), and ternary-metal MnCoNi (MCN–RuO_2) systems synthesized under identical annealing and acid-leaching conditions.

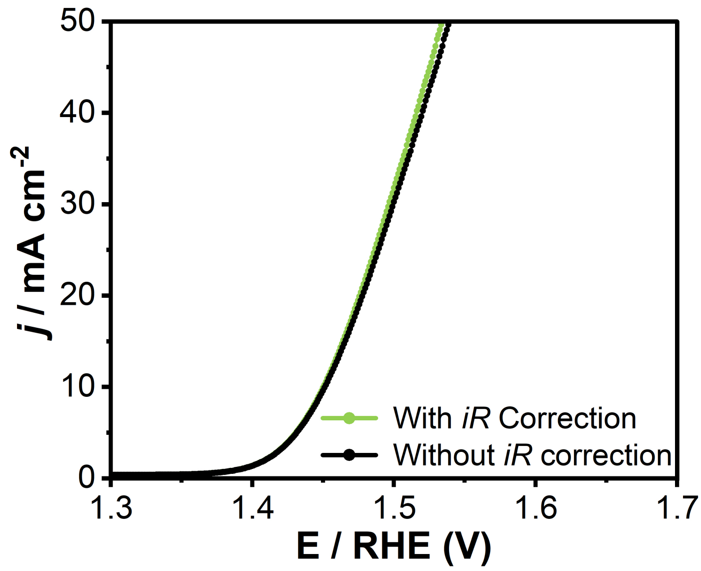

Figure S8. LSV curves of MCN–RuO_2 with and without 85% iR correction, illustrating the intrinsic catalytic activity of the synthesized material.

Table S2. Electrochemical impedance spectroscopy (EIS) parameters derived from equivalent circuit fitting for MCN–RuO_2 and benchmark catalysts in 1 M KOH. MCN–RuO_2 exhibits the lowest charge-transfer resistance.

Samples	R_s (Ω cm^2)	$\mathbf{R_{ct}}$ (Ω cm^2)	CPE	
			P	N
MCN–RuO_2	9.84	**20.9**	0.00797	0.91
RuO_2–Control.	9.75	**95.98**	0.00108	0.91
RuO_2	10.8	**124**	0.00283	0.86
IrO_2	9.80	**1095**	0.00299	0.91

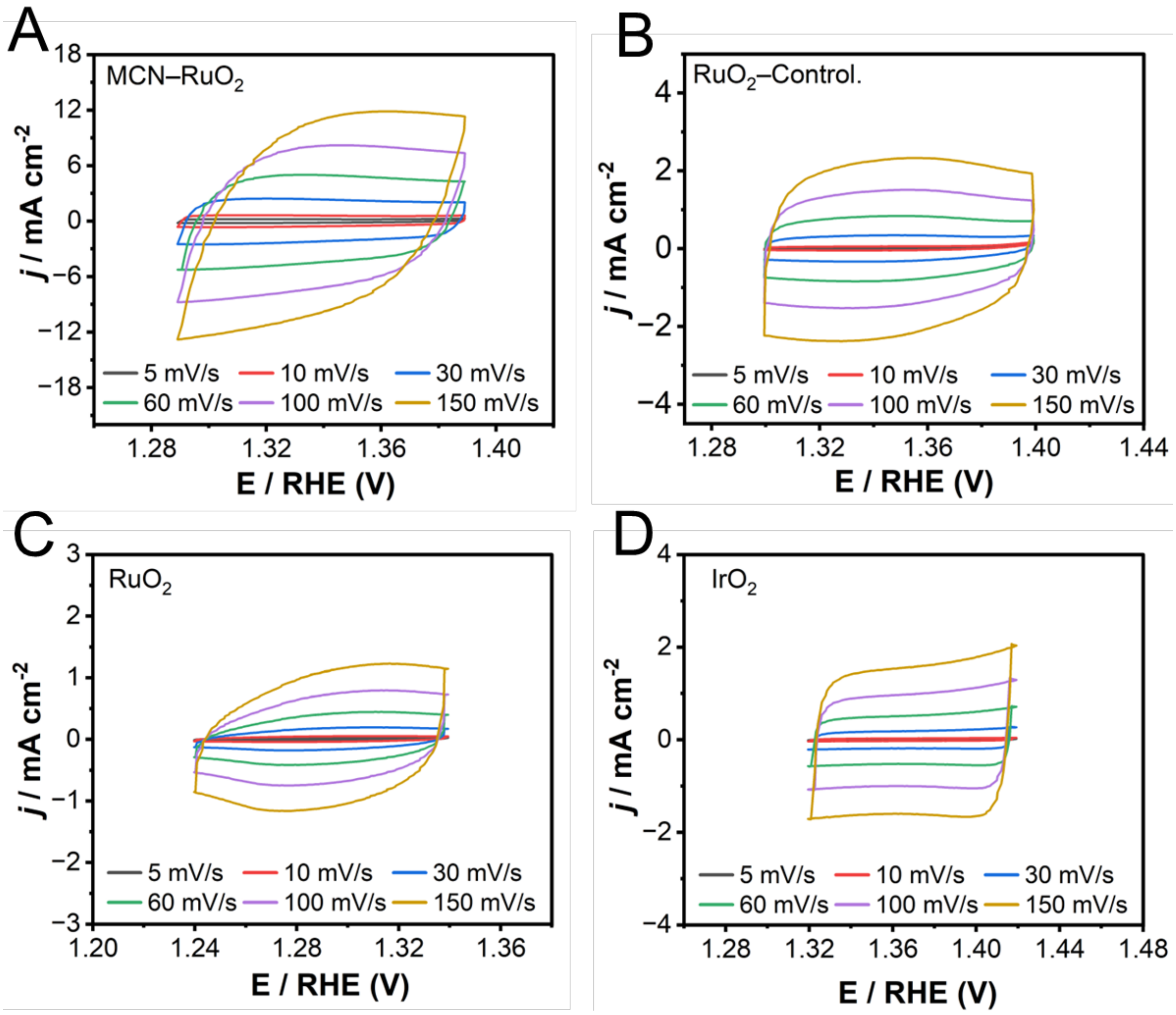

Figure S9. Cyclic voltammetry (CV) curves at multiple scan rates (5–150 mV s^{-1}) for MCN–RuO_2 and benchmark catalysts in 1 M KOH.

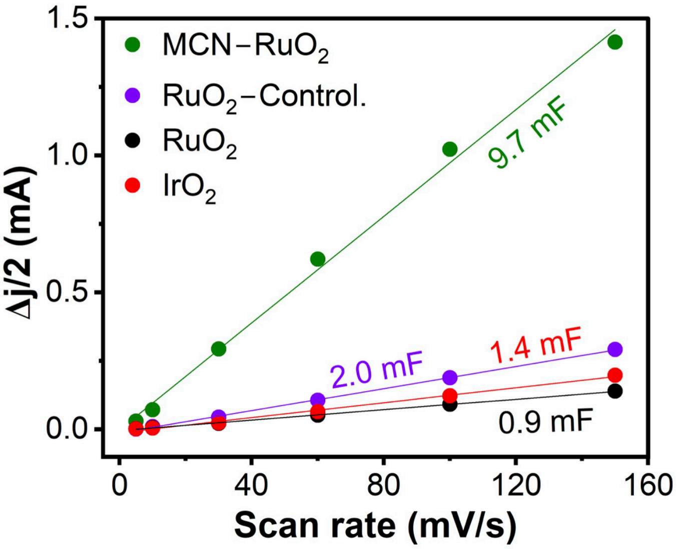

Figure S10. Extracted double-layer capacitance (C_{dl}) values from **Figure S11** used to calculate electrochemically active surface area (ECSA), with MCN–RuO_2 exhibiting the highest C_{dl}.

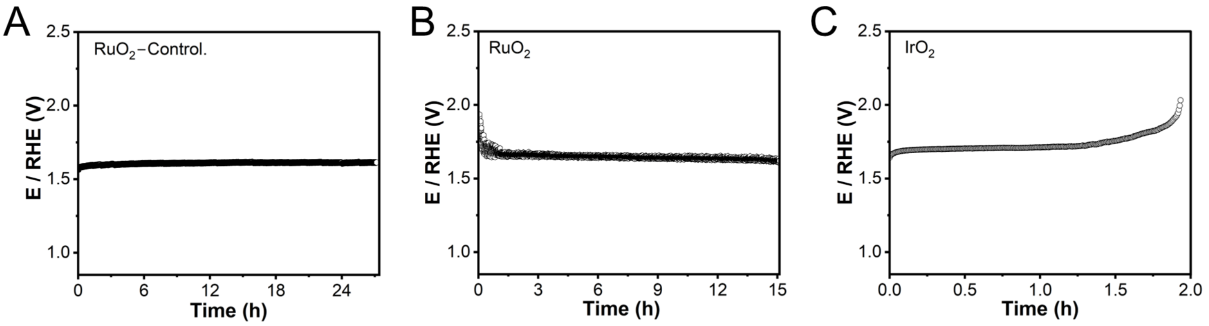

Figure S11. Chronopotentiometry curves of (A) RuO_2 control sample, (B) commercial RuO_2, and (C) commercial IrO_2 in 1 M KOH. The synthesized samples show significantly enhanced stability compared to commercial benchmarks.

Table S3. Comparison of Ru-based OER catalysts tested on glassy carbon electrodes in alkaline media, benchmarked against MCN–RuO_2 in terms of overpotential and durability

Catalyst	Overpotentials @10 mA cm^{-2} (mV)	Durability (h)	Reference
**MCN–RuO_2**	200	42	**This work**
**RuO_2/CeO_2**	350	12	[1]
**RuO_2/TiO_2**	260	13	[2]
**CoO_x/RuO_2**	230	5	[3]
**RuO_2–Fe_2O_3**	290	18	[4]
**$Ni_{1.25}Ru_{0.75}P$**	340	18	[5]
**Co-SAC/RuO_2**	200	20	[6]
**HN-Ru/RuO_2**	295	12	[7]
**A-RRO@G**	222.4	20	[8]
**RuCu**	234	12	[9]
**RuO_2 NWs**	224	20	[10]
**RuO_2/Co_3O_4**	302	N.A	[11]

N.A. represents data not available in the literature

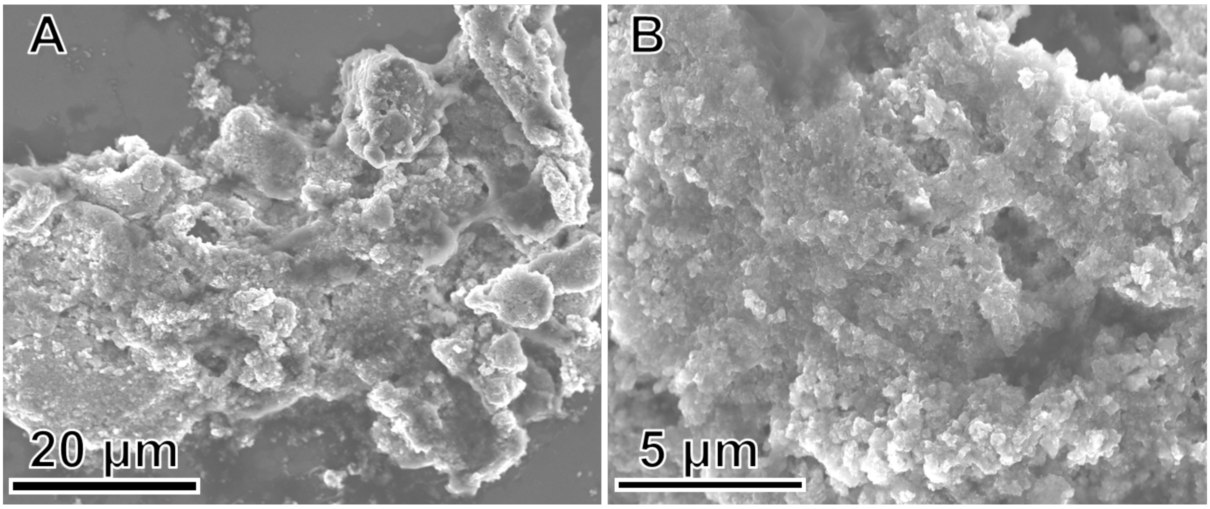

Figure S12. SEM images of MCN–RuO_2 after long-term chronopotentiometry, showing retention of nanostructured morphology.

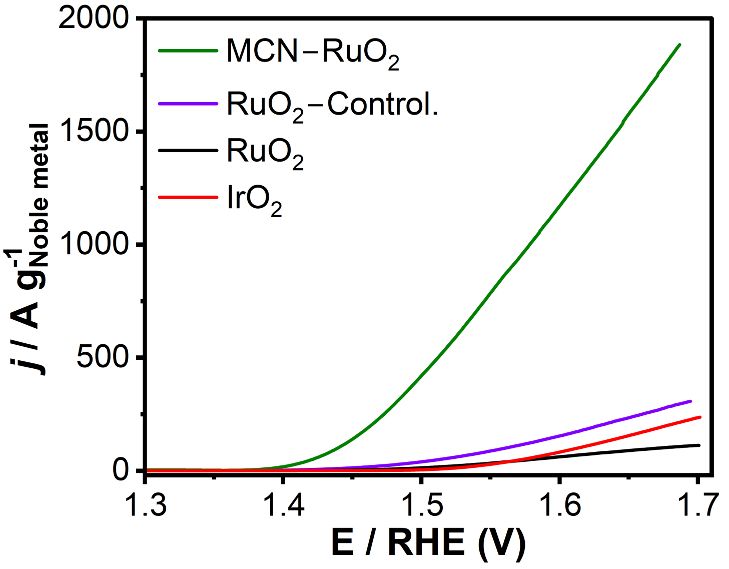

Figure S13. Noble-metal mass-normalized OER activity of MCN–RuO_2, RuO_2 control, commercial RuO_2, and IrO_2 in 1 M KOH, calculated from the iR-corrected polarization curves using the Ru or Ir content in the catalyst layer.

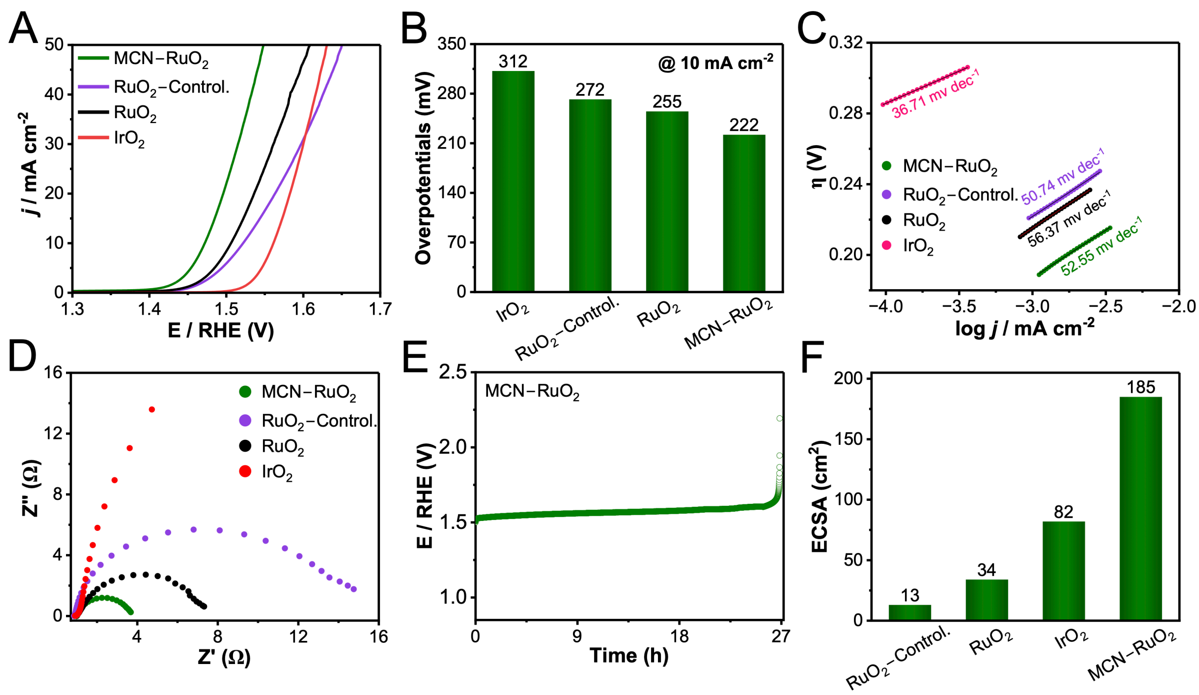

Figure S14. OER performance of MCN–RuO_2 and benchmark catalysts in acidic electrolyte (0.5 M H_2SO_4). (A) *iR*-corrected polarization curves showing enhanced activity of MCN–RuO_2 compared to commercial RuO_2 and IrO_2 as well as the RuO_2 control. (B) Comparison of overpotentials required to reach 10 mA cm^{-2}, highlighting the lowest value for the optimized catalyst. (C) Tafel plots indicating efficient reaction kinetics, with MCN–RuO_2 maintaining favorable slopes relative to benchmarks. (D) Nyquist plots from electrochemical impedance spectroscopy (EIS) at 1.50 V vs RHE. (E) Chronopotentiometry at 10 mA cm^{-2}, confirming the long-term stability of MCN–RuO_2. (F) ECSA derived from double-layer capacitance measurements, showing a significantly higher density of active sites for MCN–RuO_2.

The LSV curves (**Figure S14**) demonstrate that MCN–RuO_2 exhibits markedly enhanced OER activity in acidic electrolyte compared to commercial IrO_2 and RuO_2. At 10 mA cm^{-2}, the optimized catalyst required an overpotential of only 222 mV, significantly lower than IrO_2 (312 mV) and RuO_2 (272 mV), as well as the undoped RuO_2 control (256 mV) (**Figure 14B**). MCN–RuO_2 also displayed a lower onset potential and higher current density across the measured range. All measurements were conducted with 85% *iR*-compensation (**Figure S15**). The reaction kinetics were further examined through Tafel slope analysis (**Figure 14C**). MCN–RuO_2 exhibited a slope of 52.6 mV dec^{-1}, compared to 36.7 mV dec^{-1} for IrO_2 and higher values for RuO_2. Although slightly larger than that of IrO_2, the Tafel slope of MCN–RuO_2 is consistent with a modified reaction pathway and demonstrates efficient charge transfer at higher

current densities. Together with the LSV data, this highlights that the superior performance of MCN–RuO_2 is not limited to low-current regimes but extends across the operational range relevant to practical devices. EIS was performed at 1.50 V vs RHE (**Figure 14D**). Among all samples, MCN–RuO_2 displayed the lowest resistance (**Table S4**), confirming its superior electrical conductivity and rapid charge transport. The improved conductivity of MCN–RuO_2 can be attributed to the synergistic effects of Mn, Co, and Ni dopants, which redistribute charge density and stabilize mixed-valent states, thereby facilitating efficient electron migration across the catalyst–electrolyte interface. The durability of MCN–RuO_2 was evaluated by chronopotentiometry at 10 mA cm^{-2} (**Figure S15E**). The catalyst sustained stable operation for 27 h with minimal potential drift, surpassing commercial RuO_2 (16 h) and comparable to IrO_2 (25 h) (**Figure S16**). This extended durability positions MCN–RuO_2 among the most stable non-Ir catalysts reported on glassy carbon electrodes (**Table S5**).

Finally, the ECSA was estimated from C_{dl} measurements obtained by cyclic voltammetry at multiple scan rates (**Figure S17)**. MnCoNi–RuO_2 exhibited an ECSA of 1469 μF cm^{-2} (**Figure S18**), representing a sixfold increase relative to commercial RuO_2 and more than double that of IrO_2. Collectively, the activity, kinetic, impedance, stability, and surface-area analyses establish MnCoNi–RuO_2 as a highly efficient and durable electrocatalyst under acidic conditions.

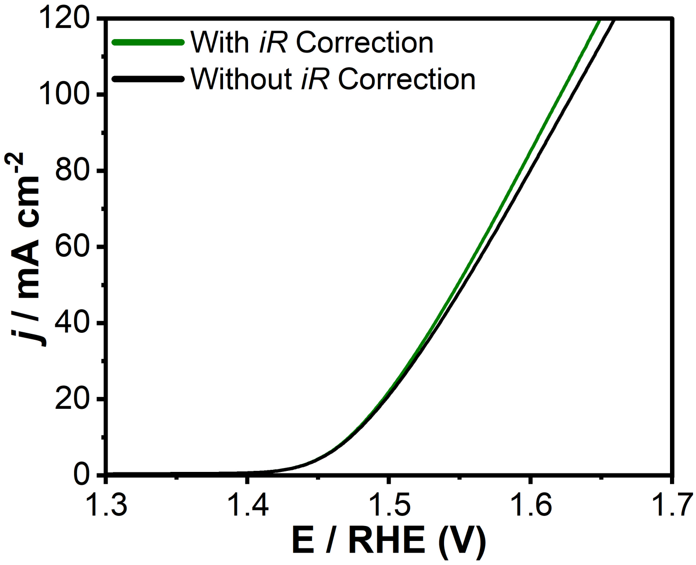

Figure S15. LSV curves of MCN–RuO_2 in 0.5 M H_2SO_4 with and without 85% *iR* correction, showing intrinsic activity improvement upon correction.

Table S4: EIS parameters of MCN–RuO_2 and benchmark catalysts in 0.5 M H_2SO_4, obtained by Randles circuit fitting.

Samples	R_s (Ω cm^2)	$\mathbf{R_{ct}}$ (Ω cm^2)	CPE	
			P	N
MCN–RuO_2	7.94	**21.6**	0.00147	0.911
RuO_2–Control.	7.02	**108**	0.00202	0.934
RuO_2	7.83	**52.9**	0.00279	0.848
IrO_2	7.84	**639**	0.00373	0.925

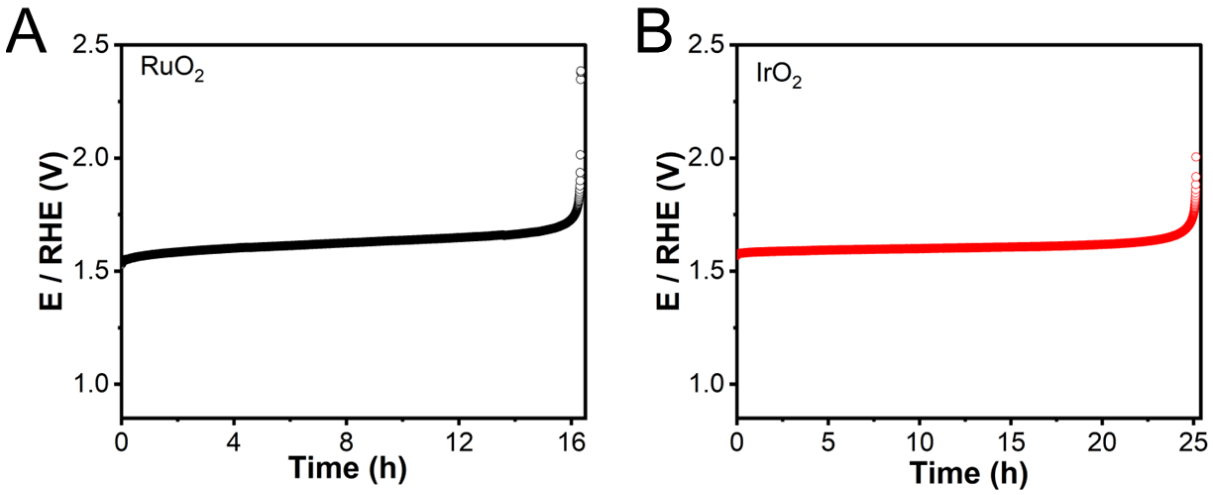

Figure S16. Chronopotentiometry of commercial RuO_2 and IrO_2 in 0.5 M H_2SO_4, highlighting faster degradation relative to MCN–RuO_2.

Table S5. Comparison of Ru-based OER catalysts tested on glassy carbon electrodes in acidic media, benchmarked against MCN–RuO_2.

Catalyst	Overpotentials @10 mA cm^{-2} (mV)	Durability (h)	Reference
**MCN–RuO_2**	220	27	**This work**
**Co-RuIr**	235	25	[12]
**RuO_2 NSs**	199	6	[13]
**$Y_2Ru_2O_{7-\delta}$**	270	8	[14]
**Mn-RuO_2**	158	10	[15]
**$RuNi_2$©G-250**	227	3	[16]
**Cu-RuO_2**	188	8	[17]
**$CaCu_3Ru_4O_{12}$**	171	24	[18]
**Ni-RuO_2**	214	20	[19]
**$Cr_{0.6}Ru_{0.4}O_2$**	178	10	[20]

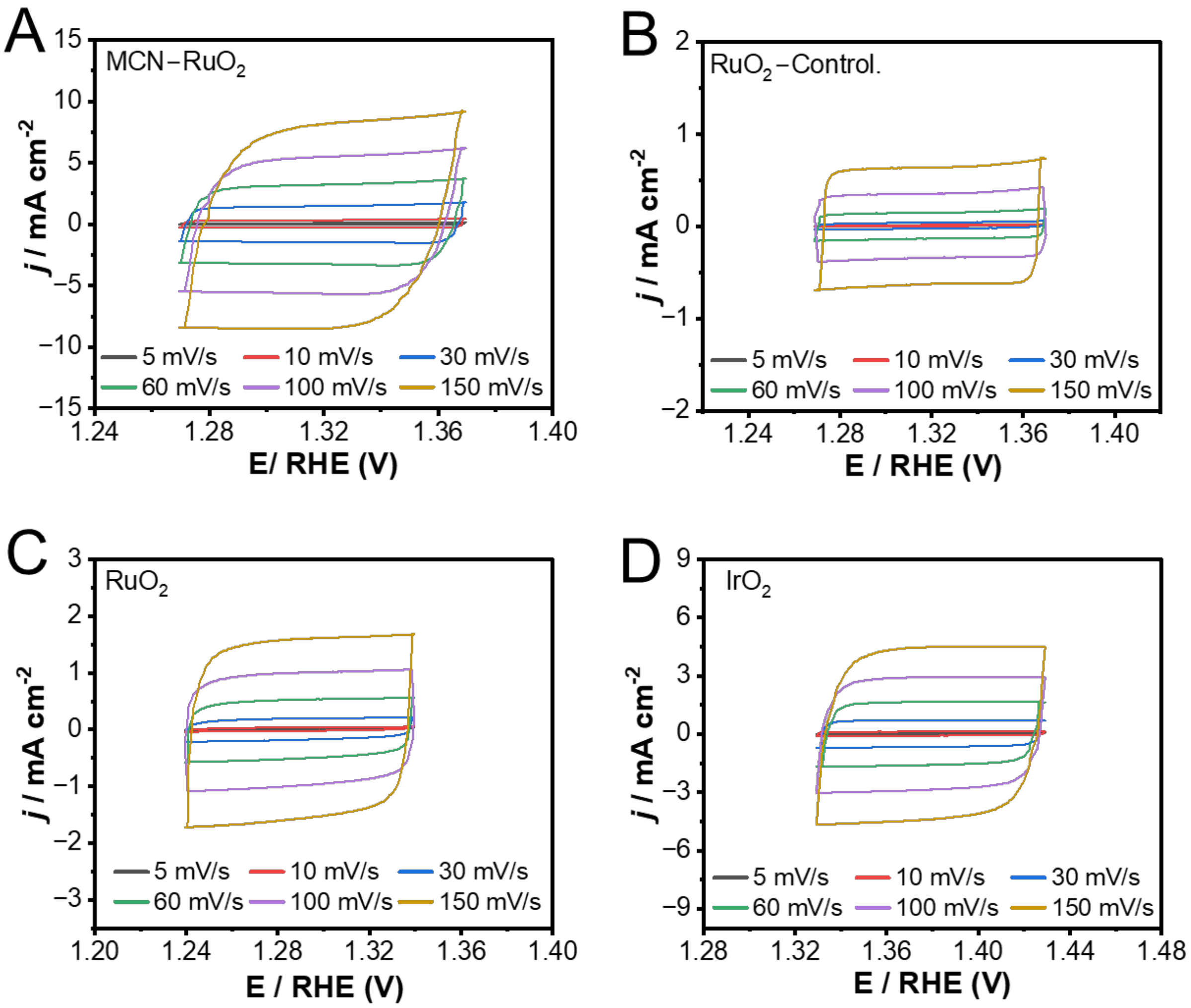

Figure S17. CV at multiple scan rates (5–150 mV s^{-1}) for MCN–RuO_2 and benchmark catalysts in 0.5 M H_2SO_4.

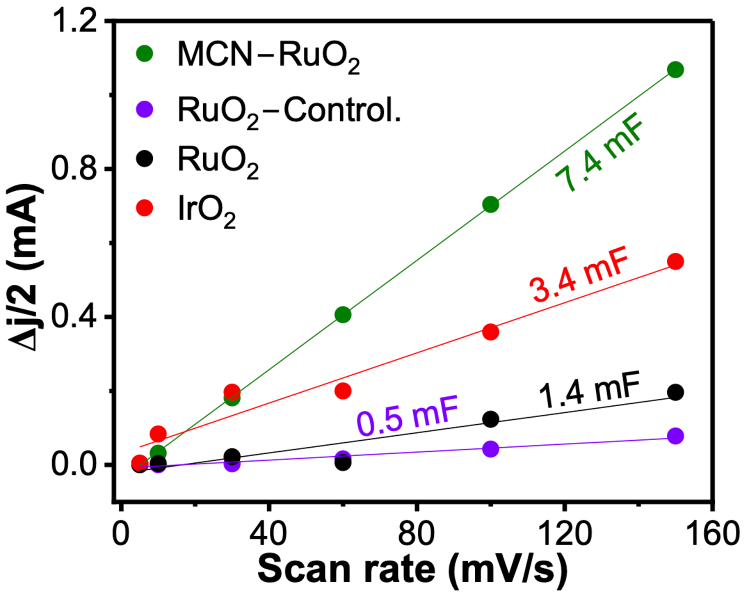

Figure S18. Extracted C_{dl} values used to calculate ECSA from Figure S16, confirming significantly higher ECSA for MCN–RuO_2.

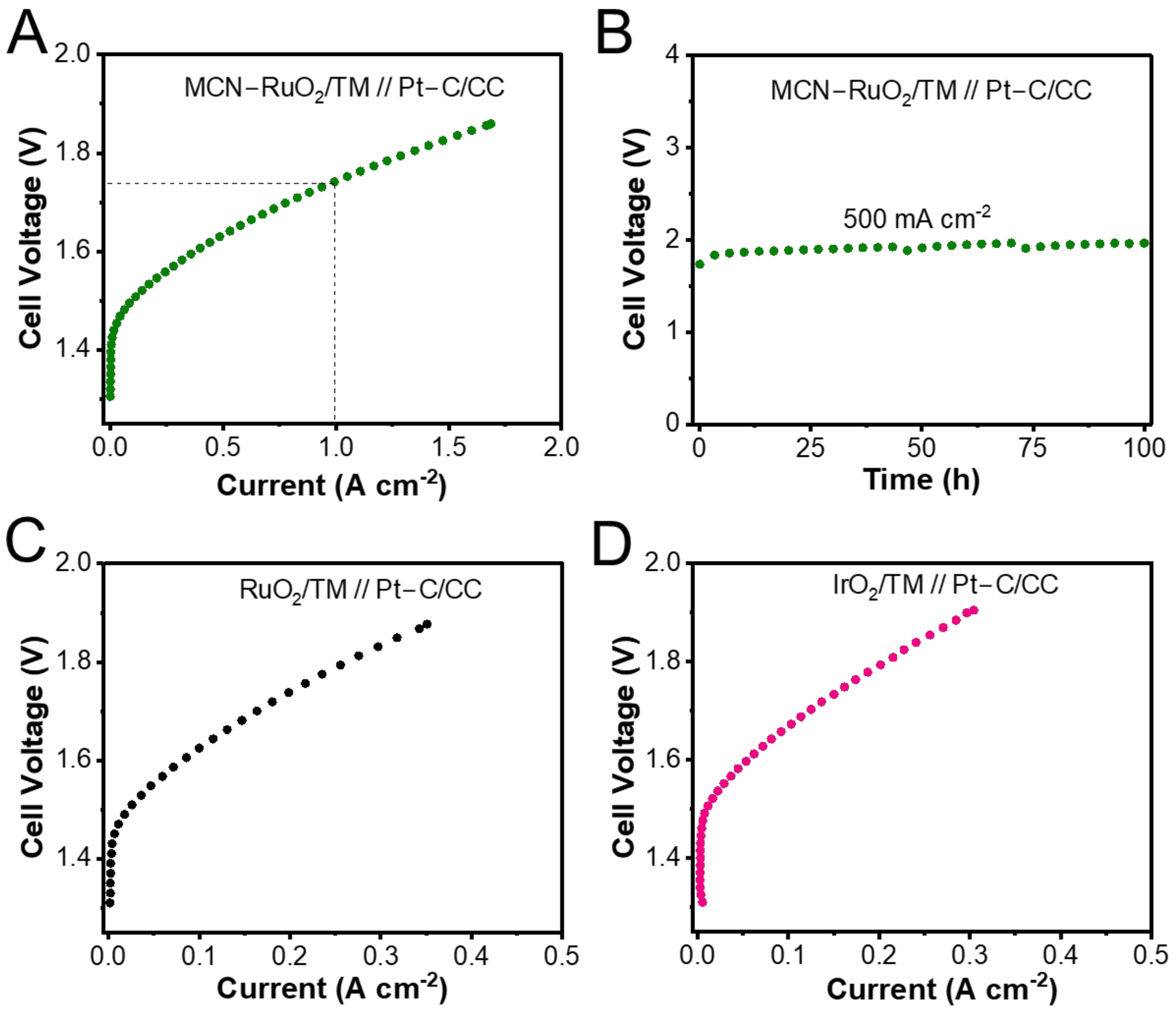

Figure S19. AEMWE single-cell performance of MCN–RuO_2 anode paired with Pt–C cathode. (a) Polarization curve using titanium mesh (TM) support, (b) stability at 500 mA cm^{-2}, and (c–d) benchmarking against commercial RuO_2 and IrO_2. MCN–RuO_2 delivers superior current density and stability under device-relevant conditions.

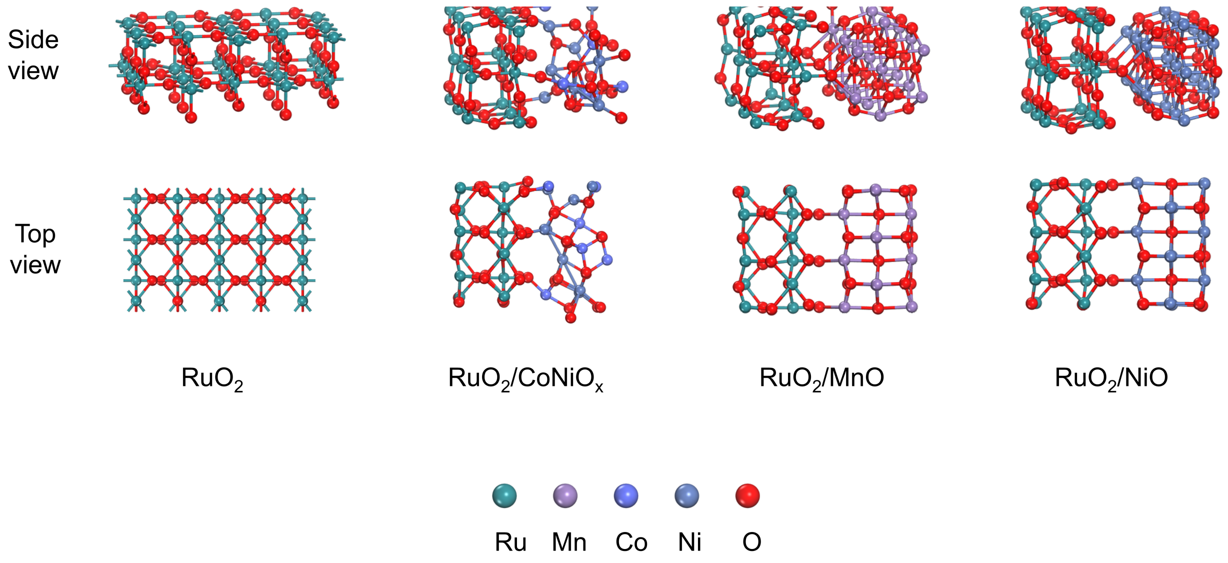

Figure S20. DFT structural models of pristine RuO_2 and heterostructures with $CoNiO_x$, MnO, and NiO, used to probe interfacial electronic interactions.

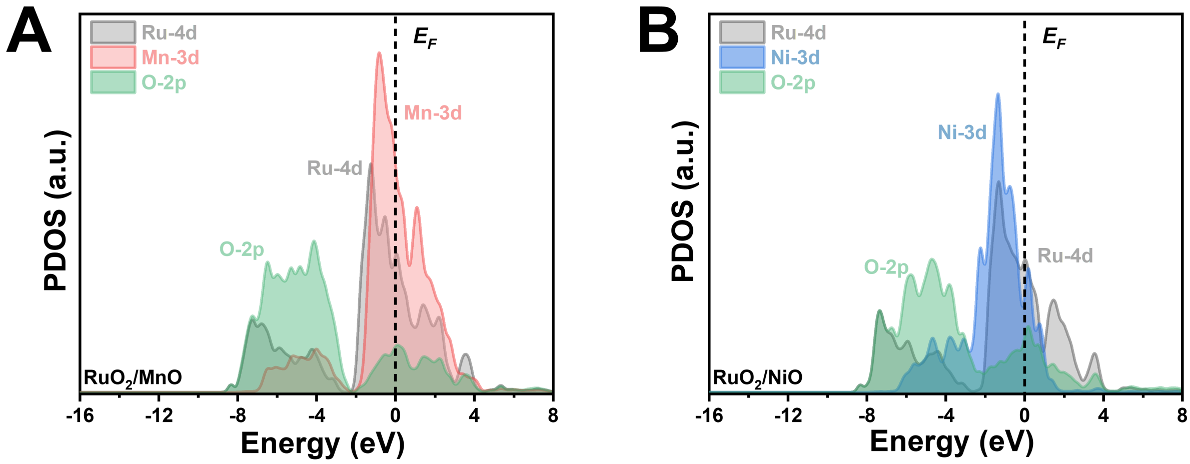

Figure S21. Projected density of states (PDOS) of RuO_2/MnO and RuO_2/NiO heterostructures, showing orbital hybridization between O-2p and transition-metal d states.

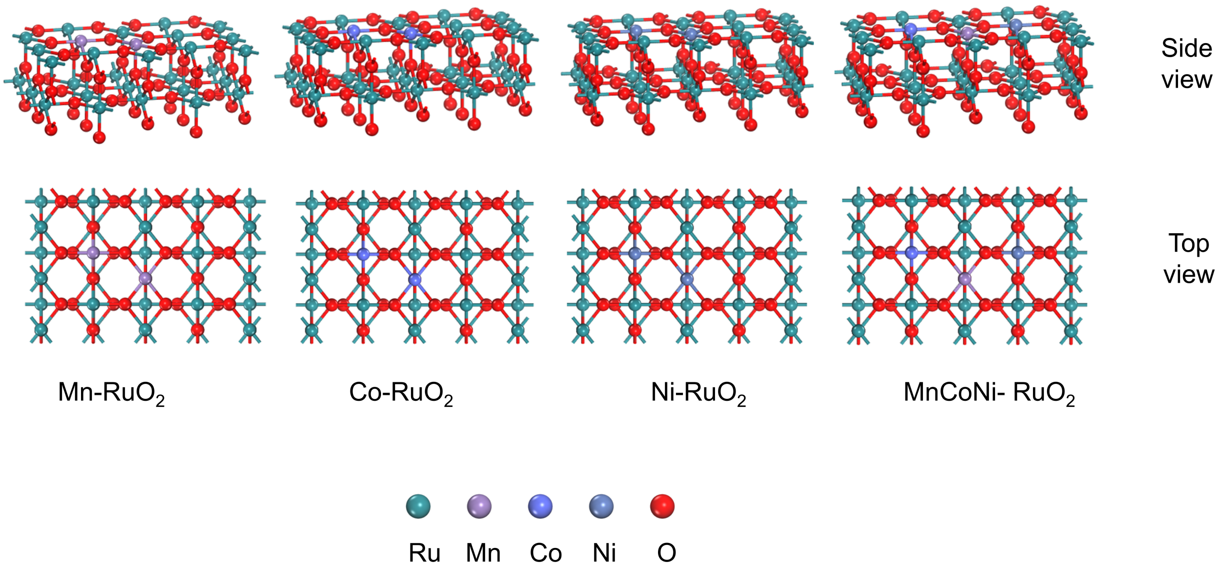

Figure S22. DFT structural models of monodoped (Mn–, Co–, Ni–RuO_2) and multi-doped (MnCoNi–RuO_2) systems, used to examine dopant effects on stability and activity.

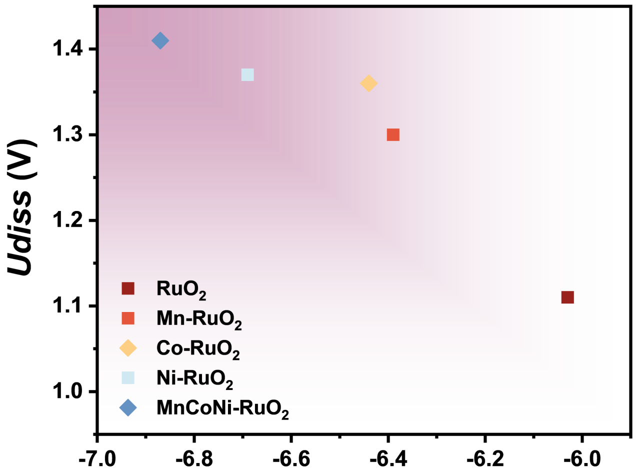

Figure S23. Binding energies and dissolution potentials (Udiss) of Ru atoms in monodoped and multi-doped RuO_2, showing enhanced stability in MnCoNi–RuO_2.

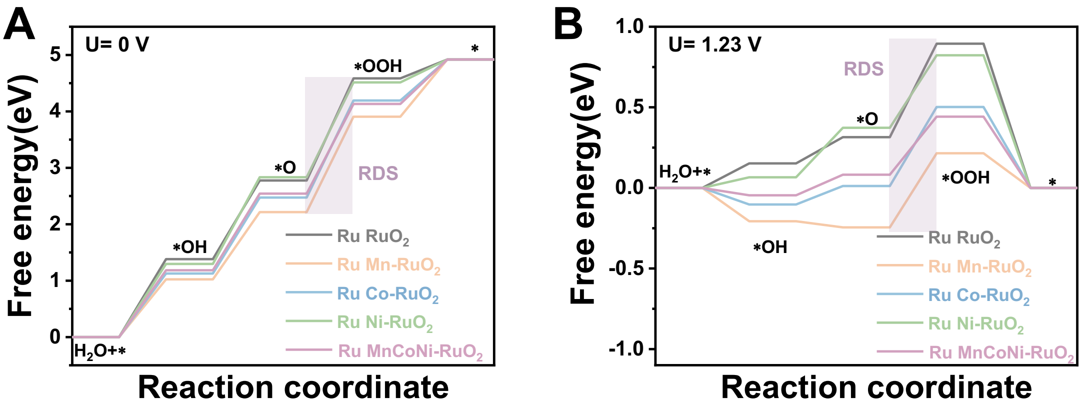

Figure S24. Free energy diagrams of OER pathways for mono-doped and multi-doped RuO_2models at (a) 0 V and (b) 1.23 V vs RHE. The MnCoNi–RuO_2 model exhibits the lowest theoretical overpotential.

Table S6. Reported performance of AEMWE devices using different anode catalysts, benchmarked against MCN–RuO_2, showing competitive activity and stability relative to state-of-the-art literature values.

Anode catalyst	MEA	AEM	Anode PTL	Cathode catalyst	Cathode PTL	Performance Test			Stability Test				References
						T [^oC]	V_{cell} [V]	J [A/cm^2]	T [^oC]	J [A/cm^2]	Time hr.	dE / dt [mV/h]	
MnCoNi-RuO_2	CCS	Pip	NF	Pt-C	CC	60	1.71	1	40	0.5	100	1.35	**This Work**
MnCoNi-RuO_2	CCS	Pip	TM	Pt-C	CC	60	1.74	1	40	0.5	100	1.06	**This Work**
IrO_2	CCS	Pip	TM	Pt-C	CC	50	1.8	0.3	50	0.2	35	1610	[21]
Ni-Fe	CCS	IM	Ni	Ni-Fe	Ni	80	2	1.62	60	0.5	1000	1.2	[22]
IrO_2	CCM	Pip	TM	Pt-C	CC	60	1.8	2.2	60	0.5	1000	0.2	[22]
$NiFe_2O_4$	CCS	QA	SS	NiFeCo	CC	60	1.8	0.24	60	1.0	200	0.4	[23]
NiFe	CCS	QA	Pt-Ti	Pt/Ru-C	CC	80	1.8	5.3	60	0.2	100	NA	[24]
NiFe	CCS	Pip	Ni	NiFe	CC	80	1.8	1.06	80	1	547	0.15	[25]
FeCo/FeCoP	CCS	QA	Pt-Ti	PtRu/C	NF	80	1.8	2.8	80	1	100	12.22	[26]
NiFeOOH	CCS	QA	SS	Pt-C	CC	70	1.9	3.6	70	3	24	6.7	[27]
$NiCo_2O_4$	CCS	QA	NF	Pt	CC	70	1.8	0.3	50	0.3	400	0.1	[28]

Abbreviations used for AEM: Pip, PiperION membrane; IM, imidazolium-backbone membrane (e.g., Sustainion®); QA, quaternary ammonium-backbone membrane.

Supplementary references

1. Galani, S. M., Mondal, A., Srivastava, D. N. & Panda, A. B. Development of RuO2/CeO2 heterostructure as an efficient OER electrocatalyst for alkaline water splitting. *Int J Hydrogen Energy* **45**, 18635–18644 (2020).
2. KC, B. R., Kumar, D. & Bastakoti, B. P. Block copolymer-mediated synthesis of TiO2/RuO2 nanocomposite for efficient oxygen evolution reaction. *J Mater Sci* **59**, 10193–10206 (2024).
3. Zeng, L. *et al.* Accelerated oxygen evolution enabled by encapsulating hybrid CoOx/RuO2 nanoparticle with nanoporous carbon. *Appl Surf Sci* **589**, 152958 (2022).
4. Mosallaei, H. *et al.* Evaluation of HER and OER electrocatalytic activity over RuO2–Fe2O3 nanocomposite deposited on HrGO nanosheets. *Int J Hydrogen Energy* **48**, 1813–1830 (2023).
5. Downes, C. A. *et al.* Controlled Synthesis of Transition Metal Phosphide Nanoparticles to Establish Composition-Dependent Trends in Electrocatalytic Activity. *Chemistry of Materials* **34**, 6255–6267 (2022).
6. Shah, K. *et al.* Cobalt Single Atom Incorporated in Ruthenium Oxide Sphere: A Robust Bifunctional Electrocatalyst for HER and OER. *Angewandte Chemie International Edition* **61**, e202114951 (2022).
7. Cho, K., Jang, J. Y., Ko, Y. J., Myung, Y. & Son, S. U. Hollow Ru/RuO2 nanospheres with nanoparticulate shells for high performance electrocatalytic oxygen evolution reactions. *Nanoscale Adv* **6**, 867–875 (2024).
8. Zhu, X. *et al.* Rapid synthesis of an aluminum-doped ultrathin Ru x –RuO 2 heterostructure optimized through combined wet–dry microwave radiation for efficient acidic and alkaline overall water splitting. *J Mater Chem A Mater* **13**, 5091–5105 (2025).
9. Yao, Q. *et al.* Channel-Rich RuCu Nanosheets for pH-Universal Overall Water Splitting Electrocatalysis. *Angewandte Chemie International Edition* **58**, 13983–13988 (2019).
10. Yang, J. *et al.* A Universal Strategy to Metal Wavy Nanowires for Efficient Electrochemical Water Splitting at pH-Universal Conditions. *Adv Funct Mater* **28**, 1803722 (2018).

11. Guo, B. Y. *et al.* RuO2/Co3O4 Nanocubes based on Ru ions impregnation into prussian blue precursor for oxygen evolution. *Int J Hydrogen Energy* **45**, 9575–9582 (2020).

12. Shan, J., Ling, T., Davey, K., Zheng, Y. & Qiao, S. Z. Transition-Metal-Doped RuIr Bifunctional Nanocrystals for Overall Water Splitting in Acidic Environments. *Advanced Materials* **31**, 1900510 (2019).

13. Zhao, Z. L. *et al.* Boosting the oxygen evolution reaction using defect-rich ultra-thin ruthenium oxide nanosheets in acidic media. *Energy Environ Sci* **13**, 5143–5151 (2020).

14. Kim, J. *et al.* High-Performance Pyrochlore-Type Yttrium Ruthenate Electrocatalyst for Oxygen Evolution Reaction in Acidic Media. *J Am Chem Soc* **139**, 12076–12083 (2017).

15. Chen, S. *et al.* Mn-Doped RuO2 Nanocrystals as Highly Active Electrocatalysts for Enhanced Oxygen Evolution in Acidic Media. *ACS Catal* **10**, 1152–1160 (2020).

16. Cui, X. *et al.* Robust Interface Ru Centers for High-Performance Acidic Oxygen Evolution. *Advanced Materials* **32**, 1908126 (2020).

17. Su, J. *et al.* Assembling Ultrasmall Copper-Doped Ruthenium Oxide Nanocrystals into Hollow Porous Polyhedra: Highly Robust Electrocatalysts for Oxygen Evolution in Acidic Media. *Advanced Materials* **30**, 1801351 (2018).

18. Miao, X. *et al.* Quadruple perovskite ruthenate as a highly efficient catalyst for acidic water oxidation. *Nature Communications 2019 10:1* **10**, 1–7 (2019).

19. Wu, Z. Y. *et al.* Non-iridium-based electrocatalyst for durable acidic oxygen evolution reaction in proton exchange membrane water electrolysis. *Nat Mater* **22**, 100–108 (2023).

20. Lin, Y. *et al.* Chromium-ruthenium oxide solid solution electrocatalyst for highly efficient oxygen evolution reaction in acidic media. *Nature Communications 2019 10:1* **10**, 1–13 (2019).

21. Chu, X., Shi, Y., Liu, L., Huang, Y. & Li, N. Piperidinium-functionalized anion exchange membranes and their application in alkaline fuel cells and water electrolysis. *J Mater Chem A Mater* **7**, 7717–7727 (2019).

22. Chen, N. *et al.* High-performance anion exchange membrane water electrolyzers with a current density of 7.68 A cm−2 and a durability of 1000 hours. *Energy Environ Sci* **14**, 6338–6348 (2021).

23. Liu, Z. *et al.* The effect of membrane on an alkaline water electrolyzer. *Int J Hydrogen Energy* **42**, 29661–29665 (2017).

24. Li, D. *et al.* Highly quaternized polystyrene ionomers for high performance anion exchange membrane water electrolysers. *Nature Energy 2020 5:5* **5**, 378–385 (2020).
25. Zheng, Y. *et al.* Anion Exchange Ionomers Enable Sustained Pure-Water Electrolysis Using Platinum-Group-Metal-Free Electrocatalysts. *ACS Energy Lett* **8**, 5018–5024 (2023).
26. Choi, G. H. *et al.* High-valent metal site incorporated heterointerface catalysts for high-performance anion-exchange membrane water electrolysers. *Applied Catalysis B: Environment and Energy* **333**, 122816 (2023).
27. Park, J. E. *et al.* Three-Dimensional Unified Electrode Design Using a NiFeOOH Catalyst for Superior Performance and Durable Anion-Exchange Membrane Water Electrolyzers. *ACS Catal* **12**, 135–145 (2022).
28. Schauer, J., Hnát, J., Brožová, L., Žitka, J. & Bouzek, K. Anionic catalyst binders based on trimethylamine-quaternized poly(2,6-dimethyl-1,4-phenylene oxide) for alkaline electrolyzers. *J Memb Sci* **473**, 267–273 (2015).
